# Impact of low-intensity heat events on mortality and morbidity in regions with hot, humid summers: a scoping literature review

**DOI:** 10.1007/s00484-022-02243-z

**Published:** 2022-01-20

**Authors:** Melanie Strathearn, Nicholas J. Osborne, Linda A. Selvey

**Affiliations:** School of Public Health, University of Queensland, Brisbane, QLD Australia

**Keywords:** Heatwave, Mortality, Morbidity, High temperature

## Abstract

The objective of this study is to determine the impacts of low-intensity heat on human health in regions with hot, humid summers. Current literature has highlighted an increase in mortality and morbidity rates during significant heat events. While the impacts on high-intensity events are established, the impacts on low-intensity events, particularly in regions with hot, humid summers, are less clear. A scoping review was conducted searching three databases (PubMed, EMBASE, Web of Science) using key terms based on the inclusion criteria. We included papers that investigated the direct human health impacts of low-intensity heat events (single day or heatwaves) in regions with hot, humid summers in middle- and high-income countries. We excluded papers written in languages other than English. Of the 600 publications identified, 33 met the inclusion criteria. Findings suggest that low-intensity heatwaves can increase all-cause non-accidental, cardiovascular-, respiratory- and diabetes-related mortality, in regions experiencing hot, humid summers. Impacts of low-intensity heatwaves on morbidity are less clear, with research predominantly focusing on hospitalisation rates with a range of outcomes. Few studies investigating the impact of low-intensity heat events on emergency department presentations and ambulance dispatches were found. However, the data from a limited number of studies suggest that both of these outcome measures increase during low-intensity heat events. Low-intensity heat events may increase mortality. There is insufficient evidence of a causal effect of low-intensity heat events on increasing morbidity for a firm conclusion. Further research on the impact of low-intensity heat on morbidity and mortality using consistent parameters is warranted.

**Supplementary Information:**

The online version contains supplementary material available at 10.1007/s00484-022-02243-z.

## Introduction

With anthropogenic climate change, the global average temperature has rapidly risen above pre-industrial levels with the past decade, 2010–2019 being the warmest on record (World Meteorological Organization 2020; Intergovernmental Panel on Climate Change 2021). Climate change has been associated with an increase in the frequency of heatwaves worldwide, with record breaking heat events in 2019 occurring in large parts of Europe, Asia and Australia (World Meteorological Organization 2020). The rise in temperatures is projected to continue to increase, and heatwaves are very likely to occur more often and with an increase in intensity and duration (Intergovernmental Panel on Climate Change 2015). The definition of a heatwave varies by location, but most definitions include reference to the number of days affected (usually two or more) and a temperature threshold, which is usually relative to the normal temperature range of that location (Anderson and Bell 2011).

One of the ways in which global increases in temperatures affect humans is through negative health impacts, including through the increased frequency and severity of extreme weather events (Watts et al. 2015). Health impacts from extreme weather events are widespread. Although the focus tends to be on more structurally damaging weather events, heatwaves kill more than bushfires, cyclones, earthquakes, floods and severe storms combined (Coates et al. 2014). Numerous studies have highlighted the increase in mortality during significant heat events (Wang et al. 2018, 2019; Murage et al. 2017). Increases in morbidity have also been highlighted by various studies (Liss et al. 2017; Jegasothy et al. 2017; Zuo et al. 2015). Health problems can be directly associated with extreme heat via the onset of heat-related medical conditions such as heat rash, heat oedema, heat syncope, heat cramps, heat exhaustion and life-threatening heatstroke (World Meteorological Organization and World Health Organization 2015). Heat-related events can also be associated with a higher risk of dehydration and associated issues such as renal failure (Hughes et al. 2016), and the exacerbation of chronic diseases like cardiovascular and respiratory disease (World Meteorological Organization and World Health Organization 2015). While anyone can be affected by heat-related illness, some populations have an increased risk. These include those with lower socio-economic status, the elderly, young children and babies, some underlying medical conditions, and people working in situations where they are more likely to be exposed to heat. Some medications also increase the likelihood of heat-related illness (Hughes et al. 2016).

Due to the increased rate of morbidity associated with severe heat events, there is often a substantial strain on health services (Liss et al. 2017; Jegasothy et al. 2017; Zuo et al. 2015), and this leads to a substantial economic burden on often already strained health systems (Wondmagegn et al. 2019). In March 2019, the greater Brisbane area experienced unseasonal hot temperatures for most of the month. Brisbane experienced the highest monthly mean maximum temperature for March in 2019 (31.2 °C), and 21 days in March 2019 had a maximum temperature of 30 °C or over. In addition, there were two consecutive periods of maximum temperatures over 30 °C in March 2019, one for 11 days (peaking at 37.7 °C) and the other 2 days later for 9 days (peaking at 34.4 °C) (Australian Bureau of Meteorology 2021). During this month, South-East Queensland hospitals were stressed, and it is possible that prolonged and unseasonal heat may have contributed to this stress, even though the heat was not extreme (Cameron and Miles 2019). While the number of hospitalisations and other measures of morbidity would be useful information, these data are not publicly available.

Heatwave intensity has been shown to be a significant factor on the level of health impacts of heatwaves. Heatwave intensity is generally measured by daily mean or maximum temperatures falling above a certain percentile, with the 97^th^ percentile seeming to be the trigger for a dramatic increase in hospitalisations and other health effects (Xu et al. 2018). Most studies have found that high-intensity heat events increase mortality, although the size of the impact varies between heatwave definitions and geographical region (Xu et al. 2016). However, both short-lasting and low-intensity heatwaves (heatwaves where the temperatures reach less than the 95^th^ percentile) have also been shown to be detrimental to health (Huang et al. 2018; Wang et al. 2015; Xu et al. 2018; Yang et al. 2019), and recommendations have been made for further studies focusing on low-intensity events to be conducted (Huang et al. 2018).

In addition, much of the published research on the impacts of heatwaves on health have been conducted in temperate regions (Campbell et al. 2018; Phung et al. 2016; Xu et al. 2016). In cooler climates, heat adaptation is likely to be less than in warmer climates (Jegasothy et al. 2017; Kovats and Hajat 2008). High humidity and other parameters such as wind speed and solar irradiation, which can differ in tropical and subtropical compared to temperate climates, can also increase the impacts of heat on human health even with lower temperatures (Gao et al. 2018; Jegasothy et al. 2017). There may not have been sufficient studies in humid regions to generate an understanding of whether the impact of heatwaves on human health is different in tropical/subtropical regions compared to temperate regions, particularly for low-intensity heatwaves. Given these potential gaps in research about the impact of low-intensity heatwaves on health in tropical and sub-tropical regions, we undertook a scoping literature review on the health impacts of low-intensity heatwaves in regions with hot, humid summers.

The primary aim of our study was to assess the impact of low-intensity heat events on morbidity and mortality in regions that experience hot, humid summers to inform public health and whole of government policy and planning for climate risk reduction. We have identified two specific research questions.

- What are the impacts of low-intensity heat events on mortality (including all-cause and cause-specific mortality)?
- What are the impacts of low-intensity heat events on morbidity, measured by ambulance dispatches, emergency department (ED) presentations and emergency hospital admissions?

### Materials and methods

This review followed the JBI Reviewer’s Manual guide for scoping reviews (Peters et al. 2017). As per the guide we defined our research question, inclusion and exclusion criteria and search strategy before undertaking the review. We did not, however, develop a formal a priori protocol prior to embarking on the review, because in undertaking the review we refined our research questions as the scope of the literature available became apparent. A scoping review was conducted as it was unclear if there was enough literature to support a systematic review and we could not formulate a question of appropriate precision for a systematic review. A scoping review allowed us to understand the research conducted in tropical/subtropical settings, while at the same time evaluating the different methodological styles and definitions of heatwave used by the authors.

## Search strategy and inclusion criteria

We conducted a literature search using three databases (PubMed, EMBASE, Web of Science). Key search terms included ‘prolonged heat’, heatwave, ‘low-intensity’, mortality, morbidity and a number of variations of these terms (Table S1).

We developed a number of key inclusion criteria prior to conducting the review (Table S1). Included papers focused on the direct human health impacts from low-intensity heat events in regions that experience hot, humid summers in high- and middle-income countries. The search strategy did not specify geographical region, but articles describing studies from places not experiencing hot, humid summers were excluded during the article screening stage. We initially defined these as geographical areas classified as Cwa (monsoon-influenced humid subtropical climates), Cfa (humid subtropical climates) and Dwa (monsoon-influenced hot-summer humid continental climates) in the Köppen climate classification (Chen et al. 2013). However, because of the limited number of articles, this was expanded to include tropical regions with hot, wet summers (Af (tropical rainforest climates), Am (tropical monsoon climates) and Aw (tropical savanna climates)). The included health impacts were mortality (all-cause and/or cause-specific) and morbidity (measured by health system utilisation: ambulance callouts, emergency department visits and hospital admissions). The definition of low-intensity heat events was either a single-day or a multiday event over a temperature threshold between the 85 and 92.5^th^ percentile. The searches were undertaken in May 2020. We did not apply a date filter to our search and excluded articles not written in English, as we did not have the capacity for translation. Full inclusion and exclusion criteria and details of search strings used can be found in Table S1.

The final search string was: (morbidity OR ‘hospital admissions’ OR ‘hospital presentations’ OR ambulance OR ‘emergency department’ OR ‘emergency admissions’ OR ‘emergency presentations’ OR mortality) AND (90^th^ OR 92.5^th^ OR 95^th^ OR ‘low intensity’ OR ‘excess heat factor’) AND (‘prolonged heat’ OR heatwave OR ‘heat index’ OR humidex OR ‘extreme heat’ OR ‘excess heat factor’ OR ‘warm spells’ OR ‘humidity’[MeSH Terms] OR ‘humidity’[All Fields] OR ‘Temperature/adverse effects’[Mesh] OR ‘Extreme Heat’[Mesh] OR ‘daily temperature*’ OR ‘ambient temperature’).

## Study selection and data extraction and analysis

We exported results from all database searches into EndNote vX9 (Clarivate Analytics, London, UK), and duplicates were removed. We then exported the remaining papers into Covidence Systematic Review Management (Veritas Health Innovation 2019). Two of us (MS and LS) screened titles and abstracts of all papers. We discussed conflicts between our decisions until reaching consensus. In the second stage of the screening process one of us (MS) screened the full text of all included papers. We categorised papers into three groups: those that were clear to MS that they met the inclusion/exclusion criteria, those that were clear to MS that they did not meet the inclusion/exclusion criteria and those that were less clear to MS. We removed papers that clearly did not meet the inclusion criteria, and all researchers discussed those that were less clear to make a decision. We included all remaining papers after the full-text screening in the final review.

We extracted key data from all included papers. The extracted data included: country/region of study, population, years of study, heat threshold (the temperature above which was considered to be the exposure), comparator threshold (the temperature range used for a comparison of morbidity or mortality), potential confounders controlled for, number of heat events, number of health events (for example number of deaths or hospitalisations), lags in days and outcomes.

### Results

**Fig. 1:**
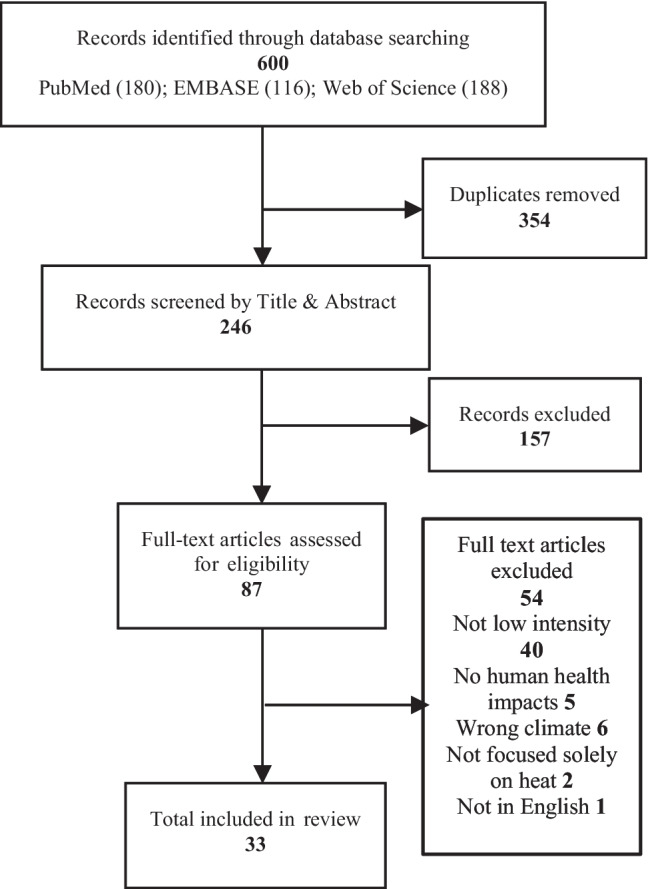
PRISMA flow diagram of literature selection process

We identified 600 articles from the three databases, with 246 articles remaining after removal of duplicates. Eighty-seven articles remained after title and abstract screening, and after reviewing the full-text articles, 54 were excluded (Fig. 1). Of the excluded articles, 40 did not investigate low-intensity heatwaves, five did not assess human health impacts, six were not in subtropical/tropical regions, two were not solely focused on the impacts of heat and one was not written in English. We included a total of 33 papers in this review (Fig. 1).

Within the results, it is important to note different methodologies used between studies (Table S2), which is likely to impact the effect size found in the studies. For heat thresholds, some studies used percentiles derived from daily temperatures across full years, while others used percentiles derived from daily temperatures from part of the years (like ‘warm season’ or ‘hot season’). Temperature thresholds for comparison also varied between studies with some comparing mortality from low-intensity heatwaves to non-heatwave days (days when a heatwave is not occurring), while other studies used temperature percentile thresholds as comparators, such as 50^th^ or 75^th^ percentiles. Minimum mortality temperature (MMT) (the temperature at which the lowest mortality occurs) and optimal morbidity temperature (the temperature at which the lowest morbidity occurs) were also used as comparators for single-day events in some studies. Lag periods also varied greatly between studies, ranging from measuring same day risk (lag 0) to up to 28 days after the event (lag 28). Data analysis methods and controls for potential confounding variables (such as air pollution and humidity) also varied between studies. Full details about the variables included in each study can be found in Table S2.

## Impact of low-intensity heat on mortality

Of the 33 papers, a total of 18 investigated the impact of low-intensity heat on mortality. The majority of studies were conducted in Asia (15) (Ban et al. 2017; Chen et al. 2013; Heo et al. 2019; Huang et al. 2018; Kim et al. 2015; Lee et al. 2016; Nori-Sarma et al. 2019; Seposo et al. 2017; Son et al. 2011; Tian et al. 2013; He et al. 2020; Yang et al. 2015, 2016b, 2019; Yin et al. 2018), with the remainder conducted in Australia (two) (Tong et al. 2014, 2015), and the USA (one) (Kent et al. 2014). Temperature thresholds ranged from 85^th^ to 92.5^th^ percentile for single-day heat events, and from 90^th^ to 92.5^th^ for heatwaves of ≥ 2 days, ≥ 3 days, ≥ 4 days and ≥ 7 days. Effects of heat ranged from same day effects and up to 28 days after the heat event.

Point estimates of relative risks for the impact of low-intensity heat events on all-cause non-accidental mortality were all above one but were not always statistically significant, ranging from 1.016 (0.908, 1.032) for heatwaves of ≥ 2 days at ≥ 90^th^ percentile compared to non-heatwave days in China (Yin et al. 2018) to 1.169 (1.131, 1.208) for heatwaves of ≥ 3 days at 90–93^rd^ percentiles compared to non-heatwave days in Thailand (Table 1). This latter estimate was for cumulative impact over ‘optimal’ lag periods (actual lags not shown). Province-specific estimates varied between regions in Thailand (Huang et al. 2018). For sub-regions in China that experience hot, humid summers, not all point estimates were greater than one. Of the 37 counties in 10 cities with hot, humid summers evaluated by Ban et al. (2017), point estimates for mortality that were significantly greater than one were found in only five, and a further 14 had non-significant point estimates greater than one (Ban et al. 2017). The study by Yin et al. (2018), which was conducted over 272 cities in China, only found a significant positive association between heatwaves and mortality for the 98 cities located in regions with Köppen classifications Dwa and Dwc (monsoon-influenced subarctic climate) for heatwaves of ≥ 2 days at ≥ 90^th^ percentile, ≥ 4 days at ≥ 90^th^ percentile and ≥ 2 days at ≥ 92.5 percentile. There was no significant association between low-intensity heatwaves and mortality for the 144 cities located in regions with Köppen classifications of Cwa, Cfa, Am or Aw (Yin et al. 2018). In the study of the association between heatwaves and mortality in 31 Chinese provincial capital cities by Yang et al. (2019), there were 23 cities with hot, humid summers. A statistically significant positive association between heatwaves and mortality for heatwaves ≥ 92^nd^ percentile was found in 13 of the 23 cities (Yang et al. 2019).

**Table 1:** Risk for all-cause mortality in low-intensity heatwaves—relative risk (95% CI)

Location	Köppen climate classification	Years of study	Heat threshold (percentile)	Comparator threshold	Single-day heat event	Heatwave of ≥2 days	Heatwave of ≥3 days	Heatwave of ≥4 days
Australia, Brisbane (Tong et al. 2015)	*Cfa*: humid subtropical climates	1988–2009, warm season	90^th^	Non-heatwave day		1.08 (1.05, 1.11)		
			92.5^th^			1.09 (1.06, 1.13)		
Australia, Sydney (Tong et al. 2015)	*Cfa*: humid subtropical climates	1988–2009, warm season	90^th^	Non-heatwave day		1.06 (1.04, 1.08)		
			92.5^th^			1.08 (1.06, 1.11)		
Australia, Brisbane (Tong et al. 2014)	*Cfa*: humid subtropical climates	1988–2009, hot season	90th	Non-heatwave day		1.04 (1.01, 1.07)		
Australia, Sydney (Tong et al. 2014)	*Cfa*: humid subtropical climates	1988–2009, hot season	90th	Non-heatwave day		1.04 (1.03, 1.06)		
China, 43 counties in 12 cities (Ban et al. 2017)	> 1: 38 counties with hot, humid summers	2013–2015	90^th^	75^th^ percentile	1.046 (1.034, 1.057) (overall estimate, Lag 0–2)			
China, 272 cities (Yin et al. 2018)	> 1: 242 cities with hot, humid summers	2013–2015	90^th^	Non-heatwave day		1.016 (0.980, 1.032)	1.005 (0.850, 1.025)	1.015 (0.980, 1.028)
			92.5^th^			1.016 (0.980, 1.032)	1.020 (1.001, 1.037)	1.024 (1.005, 1.043)
China, 31 provincial capital cities (Yang et al. 2019)	> 1: 23 cities with hot, humid summers	2007–2013	90^th^	Non-heatwave day		1.030 (1.020, 1.050)	1.040 (1.025, 1.070)	1.048 (1.027, 1.075)
			92.5^th^			1.044 (1.026, 1.065)	1.060 (1.030, 1.089)	1.060 (1.030, 1.090)
India, Mumbai (Nori-Sarma et al. 2019)	*Aw*: tropical savanna climates*, summer humidity—0–80%*	2000–2012	90^th^	Non-heatwave day		Significantly greater than 1 and less than 1.1	Significantly greater than 1 and less than 1.1	Significantly greater than 1 and less than 1.1
			92.5^th^			Significantly greater than 1 and less than 1.1	Significantly greater than 1 and less than 1.1	Significantly greater than 1.1 and less than 1.2
Philippines (Seposo et al. 2017)	> 1, predominantly Am: tropical monsoon climates, Af: tropical rainforest climates	2006–2010	90^th^	75^th^ percentile		1.125 (1.047, 1.209)		1.135 (1.031, 1.251)
South Korea (Lee et al. 2016)	> 1, predominantly Dwa: monsoon-influenced hot-summer humid continental climate	1992–2012	AT 90^th^	Non-heatwave day		1.037 (0.972, 1.106)	1.047 (0.985, 1.114)	1.058 (1.002, 1.118)
South Korea (Heo et al. 2019)	> 1, predominantly Dwa: monsoon-influenced hot-summer humid continental climates	2011–2014 warm season	WBGT_max_ 90^th^	Non-heatwave day	1.035 (1.005–1.066), Lag 0–1	1.021 (1.006–1.036)		
South Korea, Seoul (Kim et al. 2015)	*Dwa*: monsoon-influenced hot-summer humid continental climates	1992–2009	93^rd^	90^th^ percentile	1.030 (1.020–1.030)			
South Korea, Seoul (Son et al. 2011)	*Dwa*: monsoon-influenced hot-summer humid continental climate	2000–2007	90^th^	50^th^ percentile	1.093 (1.065, 1.122)			
Thailand, 60 provinces (Huang et al. 2018)	> 1, *Aw*: tropical savanna climates, *Am*: tropical monsoon climate, *Af*: tropical rainforest climate	1999–2008	90^th^–93^rd^	Non-heatwave day				1.169 (1.131, 1.208) (pooled cumulative effect over lag 0–21. 1.113 (1.097, 1.130) at lag 0–1
USA, Alabama (Kent et al. 2014)	*Cfa*: Humid subtropical climates	1990–2010	85^th^	Non-heatwave day	1.012 (0.999, 1.026)			
			90^th^		1.020 (1.003, 1.038)	1.037 (1.011, 1.063)		

Studies are ordered by country (alphabetical)

*AT* apparent temperature, *MMT* minimum mortality temperature, *WBGT*_*max*_ maximum Wet Bulb Globe Temperature

Mortality impacts from low-intensity heat events can occur over long lag periods, as found in a study from China which found a relative risk of mortality compared to non-heatwave days at lag 0 of 1.060 (1.030, 1.089), at cumulative lag of 0–2 days 1.09 (1.05, 1.13) and at cumulative lag of 0–10 days 1.10 (1.05, 1.15) (Yang et al. 2019). The risk of mortality during low-intensity heat events is not just confined to summer, as demonstrated by the findings of two studies using data over the same time period in Australia: one focusing on the ‘hot season’ (December–February) (Tong et al. 2014) and the other on the ‘warm season’ (November–March) (Tong et al. 2015). Both of these studies measured the relative risk of mortality during heatwave days (≥ 2 day at ≥ 90^th^ percentile of daily mean temperature in the respective ‘season’) compared to non-heatwave days (days not meeting the heatwave definition) with results in both Brisbane and Sydney demonstrating a similar level of risk during ‘warm’ and ‘hot’ seasons (relative risk Brisbane: 1.08 (1.05, 1.11); relative risk Sydney: 1.06 (1.04, 1.08) (Tong et al. 2015), relative risk Brisbane: 1.04 (1.01, 1.07); relative risk Sydney: 1.04 (1.03, 1.06) (Tong et al. 2014)) respectively (Table 1).

**Fig. 2:**
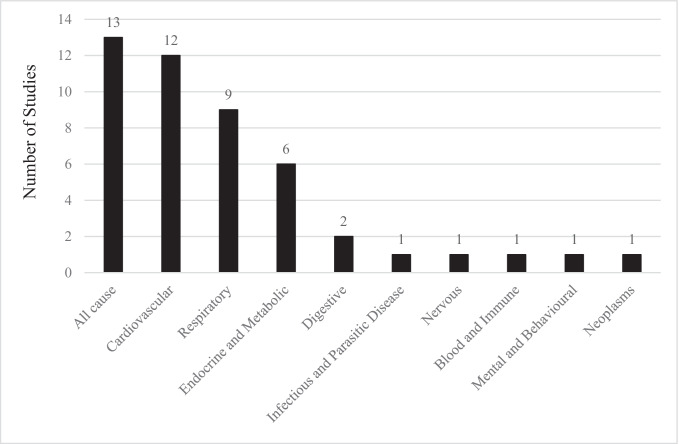
Distribution of studies including data on cause-specific mortality during low-intensity heat events

A total of 13 papers investigated the impact of low-intensity heat on mortality due to various causes. The most common causes of mortality that were investigated were cardiovascular (12 papers) (Ban et al. 2017; Chen et al. 2013; Heo et al. 2019; Huang et al. 2018; Kim et al. 2015; Lee et al. 2016; Seposo et al. 2017; Son et al. 2011; Tian et al. 2013; Yang et al. 2015, 2019; Yin et al. 2018) and respiratory mortality (nine papers) (Ban et al. 2017; Heo et al. 2019; Huang et al. 2018; Kim et al. 2015; Lee et al. 2016; Seposo et al. 2017; Son et al. 2011; Yang et al. 2019; Yin et al. 2018) (Fig. 2). There were also five papers investigating the impact of low-intensity heat events on the endocrine system, particularly diabetes (He et al. 2020; Huang et al. 2018; Kim et al. 2015; Yang et al. 2016b, 2019). The impact of low-intensity heat events on cardiovascular-, respiratory- and endocrine (particularly diabetes) system-related mortality increased the risk of mortality during low-intensity heat events due to causes in all three of these categories. Studies also investigated the impact of low-intensity heatwaves on mortality due to a range of other conditions (Fig. 2). Results from these studies indicate that there may be an increase in mortality in these conditions during low-intensity heat events (Huang et al. 2018; Kim et al. 2015). However, these results should be interpreted with caution as they have been reported in a limited number of papers and not all estimates were statistically significant (Table S2).

## Impact of low-intensity heat on morbidity

**Fig. 3:**
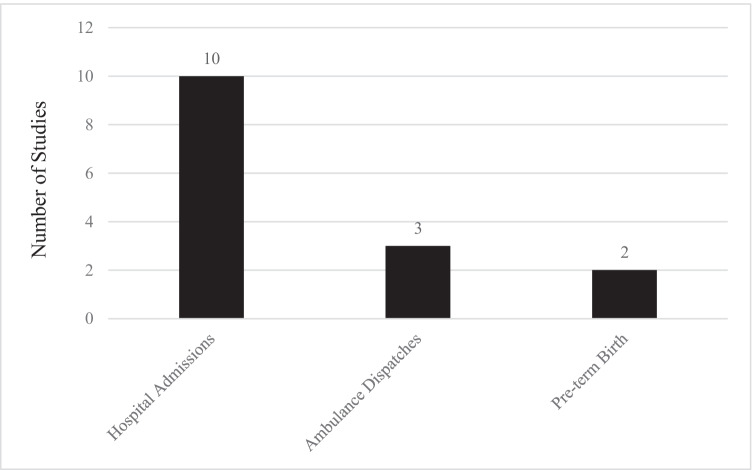
Distribution of studies on morbidity during low-intensity heat events by outcome

A total of 15 studies investigated the impact of low-intensity heat on morbidity (Table 2, Supplemental Table 2). The majority of these studies were conducted in Asia (8) (Cui et al. 2019; Ge et al. 2018; Heo et al. 2019; Kotani et al. 2018; Sun et al. 2014; Yang et al. 2016a; Yi et al. 2019; Zhang et al. 2020), while the remainder were conducted in Australia (Xu et al. 2017, 2019b, 2019c), the USA (Gronlund et al. 2014; Kent et al. 2014; Sun et al. 2019) and Brazil (Zhao et al. 2019). The most common morbidity outcome measured was hospital admissions with 10 papers reporting this outcome (Fig. 3).

**Table 2:** Risk for morbidity in low-intensity heatwaves—risk estimate (95% CI)

Location	Köppen climate classification	Years of Study	Condition	Heat threshold	Comparator threshold	Single-day heat event	Heatwave of ≥2 days	Heatwave of ≥3 days	Heatwave of ≥4 days
1814 cities in Brazil Zhao et al, 2019	> *1. Af: tropical rainforest climate, Am: tropical monsoon climate, Aw: tropical savanna climate, Cfa: humid subtropical climates, Cwa: monsoon-influenced humid subtropical climates, Cfb: temperate oceanic climates, Cwb: subtropical highland or monsoon-influenced temperate oceanic climates (71% of cases); and As: tropical dry savanna climates, BsH: hot semi-arid climates (29% of cases)*	2000–2015, five hottest months	All-cause and numerous cause-specific hospital admissions	90th and 92.5th percentiles daily mean temperature	Non-heatwave day		All cause: small (< 2.5%), significantly increased RR nationally for 90th and 92.5th percentiles	As per 2-day heatwaves	As per 2 and 3-day heatwaves
Brisbane, Australia Xu et al. 2017	*Cfa:* humid subtropical climates	2005–2015	All cause infant (< 1 year) hospital admissions	90th percentile daily mean temp	Non-heatwave day		Small, positive, non-significant RR. Adjusted for air pollutants, humidity, season, long-term trends	Small, positive, non-significant RR. Adjusted for air pollutants, humidity, season, long-term trends	As per 2 and 3-day heatwaves
Hefei City, China Cui et al. 2019	*Cfa:* humid subtropical climates	2015–2017	Cardiovascular disease, hospital admissions	90th percentile maximum	75th percentile (whole year)	RR 1.015 (0.988–1.043) Lag 0, all. RR 0.982 (0.938, 1.035), Lag 0, < 65, RR 1.081 (1.012, 1.154), Lag 0, ≥ 65			
South Korea Heo et al. 2019	> *1, predominantly Dwa: monsoon-influenced hot summer humid continental climates*	2011–2014 warm season	Emergency hospital admissions for cardiovascular disease, respiratory disease and heat disorders	90th percentile WBGT_max_	Non-heatwave day	Cardiovascular: RR 1.077 (1.013, 1.146). Respiratory: RR 0.969 (0.912, 1.029). Heat disorders: RR 1.663 (1.180, 2.343) Lag 0–1	Cardiovascular: RR 1.013 (0.963, 1.066). Respiratory: RR 1.124 (1.038, 1.217). Heat disorders: RR 2.363 (1.382, 4.038) Lag 0–1		
Shanghai, China Ge et al. 2018	*Cfa:* humid subtropical climates	2013–2015	Rheumatic heart disease, hospital admissions	90th percentile daily mean temp	0 °C	RR 2.55 (1.14, 5.73), cumulative lag 0–5, unadjusted, RR 2.70 (1.19, 6.15) adjusted PM2.5 and ozone), higher in ≥ 65			
Beijing, China Zhang et al. 2020	*Dwa*: monsoon-influenced hot summer humid continental climates	2013–2016	Chronic obstructive pulmonary disease, hospital admissions	90th percentile daily mean temp and AT	75th percentile daily mean temp and AT	RR 1.09 (0.93, 1.26), Lag 30 mean temp. RR 1.07 (0.92, 1.24), Lag 30 AT			
Brisbane, Australia Xu et al. 2019b	*Cfa:* humid subtropical climates	2005–2013	Diabetes, hospital admissions	90th percentile daily mean temp	Non-heatwave day		OR 1.09 (0.97, 1.23), Lag 0. OR 1.04 (0.93, 1.18), Lag 1. OR 0.97 (0.86, 1.10) Lag 2		
Hefei City, China Yi et al. 2019	*Cfa*: humid subtropical climates	2005–2014	Schizophrenia hospital admissions	AT 90th percentile	AT minimum admissions 3.3 °C	RR 1.062 (1.019, 1.106), Lag 0			
Brisbane, Australia Xu et al. 2019c	*Cfa*: humid subtropical climates	2005–2013	Alzheimer’s disease hospital admissions	90th percentile daily mean temp	Non-heatwave day		Non-significant OR around 1.25, Lag 0–7		
Pudong New Area, Shanghai, China Sun et al. 2014	*Cfa*: humid subtropical climates	2011–2013 warm season	All cause emergency department visits and ambulance dispatches	90th percentile daily mean temp	Non-heatwave day	RR 1.009 (0.92, 1.019), EDV 90th percentile; 1.06 (1.02, 1.10), EAD, 90th percentile	RR 1.026 (1.018, 1.035) EDV. 1.049 (1.014, 1.084) EAD	RR 1.0095 (1.002, 1.017) EDV. 1.039 (1.009, 1.071) EAD	
Fukuoka, Japan Kotani et al. 2018	*Cfa:* humid subtropical climates	2005–2012 warm season	All cause ambulance dispatches	85th percentile daily mean temp	OptRefT	RR 1.08 (1.05, 1.12), Lag 0. All ages			
Guangzhou, China Yang et al. 2016a, b	*Cwa*: humid subtropical climates	2008–2012	Renal colic emergency ambulance dispatches	90th percentile	21 °C	RR 1.92 (1.21, 3.05), Lag 0–7			
Alabama, USA Kent et al. 2014	*Cfa*: humid subtropical climates	1990–2010	Pre-term birth	85th and 90th percentile	Non-heatwave day	No significant change in RR 85th percentile. Small (< 5%) significant RR 90th percentile			
USA Sun et al. 2019	58 counties hot-humid (largely *Cfa*: humid subtropical climates) 113 counties mixed-humid (largely *Cfa*)	1989–2002	Pre-term birth	50th to 90th percentile (moderate heat)	Below 50th percentile	OR 1.26 (1.26, 1.27) pre-term births in hot-humid and OR 1.18 (1.18, 1.18) in mixed-humid zones			

Studies are ordered by the mode of presentation and by cause

*AT* apparent temperature, *OptRefT* optimum reference temperature, *WBGTmax* maximum Wet Bulb Globe Temperature, *EDV* emergency dept visits, *EAD* emergency ambulance dispatches, *RR* relative risk, *OR* odds ratio

## Hospital admissions: all ages

A total of 10 papers investigated the impact of low-intensity heat events on hospital admissions. Two of these focused on either infants (Xu et al. 2017) or older people (Gronlund et al. 2014) and will be discussed separately. The remaining eight papers investigated hospital admissions for a range of conditions across all ages. Only one of these papers investigated all-cause hospital admissions (Zhao et al. 2019), which found that there was a slight but significant increase (percent risk increase of less than 2.5) in admissions during low-intensity heat events of between 2- and 4-day duration across all regions in Brazil, and separately in two of the four regions in Brazil with hot, humid summers (Table 2) (Zhao et al. 2019).

Three papers included data on all cardiovascular-related admissions (Cui et al. 2019; Heo et al. 2019; Zhao et al. 2019), and two papers included data on all respiratory-related admissions (Heo et al. 2019; Zhao et al. 2019). All studies found an increased risk of cause-specific hospital admissions related to low-intensity heatwaves (not always statistically significant), except in Brazil, where a small, statistically significant decreased risk of cardiovascular disease-related admissions associated with low-intensity heatwaves across all regions was found (Zhao et al. 2019). Low-intensity heat events were also found to be positively associated with hospital admissions for rheumatic heart disease (RHD) (Ge et al. 2018), heat-related illness (Heo et al. 2019), schizophrenia (Yi et al. 2019) and endocrine-, genito-urinary- and skin-related disorders (Zhao et al. 2019) (Table S2). A number of studies investigated the association between low-intensity heatwaves and hospital admissions for other specific disorders but found small, or no associations. These disorders included diabetes (Xu et al. 2019b), chronic obstructive pulmonary disease (COPD) (Zhang et al. 2020), neoplasms (Zhao et al. 2019) and Alzheimer’s disease (Xu et al. 2019c) (Table S2).

## Hospital admissions: infants and children

Three studies investigated the impacts of low-intensity heat events on morbidity among infants and children. Xu et al. (2017) investigated the impact of low-intensity heatwaves on all-cause infant (< 1 year old) morbidity in Brisbane, Australia and found no statistically significant association (Table 2). One study investigated the association between low-intensity heat events and all-cause morbidity in children up to 19 years old and found a slight increase in hospital admissions during low-intensity heat events across all regions in Brazil (Zhao et al. 2019). The highest increase was in children under 4 years (around a 5% risk increase) (Zhao et al. 2019). The third study investigated the association between low-intensity heatwaves and diabetes-related hospital admissions, finding an increased risk of diabetes-related hospital admissions for children aged 0–14 years (OR 1.36 (1.04, 1.78)) (Xu et al. 2019b).

## Hospital admissions: older people

A total of eight studies investigated the impact of low-intensity heat events on older people. Two of these studies investigated all-cause hospital admissions (Gronlund et al. 2014; Zhao et al. 2019) and found only a slight increase in hospital admissions. The study by Gronlund et al. (2014) investigated the impact of low-intensity heat events on admissions for those aged ≥ 65 years and had a relative risk of 1.007 (1.005, 1.008) across all climate regions, and a small but statistically significant increased risk in New York City, but not Houston (two cities with hot, humid summers where city-specific data were available). The greatest effect in New York City was with a cumulative lag period of 0–7 days (Gronlund et al. 2014). The study by Zhao et al. (2019) reported outcomes by age groups and found that in the older population across Brazil, hospital admissions in low-intensity heat events increased with age. There was a small but statistically significant risk increase (less than 2.5%) in hospitalisations among people aged 70–79 for heatwaves of between 2 and 4 days duration at the 92.5^th^ percentile, and between a 5 and 7.5% increase among people aged 80 and over for heatwaves of between 2 and 4-day duration and at 90^th^ and 92.5^th^ percentiles (Zhao et al. 2019).

Two studies investigated hospital admissions for cardiovascular disease in adults aged ≥ 65 years (Gronlund et al. 2014; Cui et al. 2019). These studies had conflicting results with the study by Cui et al. (2019) reporting a small increase in all cardiovascular admissions (relative risk 1.081 (1.012–1.154)), while the study by Gronlund et al. (2014) reported a small decrease in cardiovascular admissions across all climate regions (relative risk of 0.987 (0.940, 0.990)).

One study investigated the impact of moderately high temperatures on hospital admissions for rheumatic heart disease in Shanghai across all ages and for people aged ≥ 65 years (Ge et al. 2018). This study found an increase in rheumatic heart disease-related hospital admissions during low-intensity heat days (compared to days of 0 °C) for all ages with a relative risk of 2.70 (1.19–6.15) with a cumulative lag of 0–5 days. For people aged ≥ 65 years, the relative risk of rheumatic heart disease-related hospital admission was between 3.04 (1.05–8.83) with a cumulative lag of 0–5 days and 4.55 (2.00–10.33) with a cumulative lag of 0–2 days. The relative risk for hospital admission for people under 65 years was positive but not significant (Ge et al. 2018). One study investigated hospital admissions related to renal disease in people aged ≥ 65 years (Gronlund et al. 2014). This study found that there was an increase in renal-related hospital admissions during days with an apparent temperature at or above the 90^th^ percentile across all climate regions in the USA with a relative risk of 1.043 (1.030, 1.056) and a cumulative lag of 0–7 days.

A number of studies investigated hospital admissions among older people for other specific disorders but found no statistically significant association. These disorders included all respiratory diseases (Gronlund et al. 2014), diabetes (Xu et al. 2019b), COPD (Zhang et al. 2020), schizophrenia (Yi et al. 2019) and Alzheimer’s disease (Xu et al. 2019c).

## Hospital admissions: maternal and peri-natal health and pre-term birth

Two studies investigated the impact of low-intensity heat events on pre-term births (Sun et al. 2019; Kent et al. 2014). One study, conducted in Alabama, USA (Kent et al. 2014), found no association between low-intensity heat events and pre-term birth. The other, utilising data from 171 counties in hot and mixed humid regions in the USA (Sun et al. 2019), found an odds ratio for pre-term births of 1.26 (1.16, 1.27) in hot-humid and 1.18 (1.18, 1.18) in mixed humid regions on days with temperatures ≥ 90^th^ percentile.

Only one study investigated hospital admissions for perinatal and maternal disorders. This study found a statistically significant increase in both perinatal admissions (relative risk between 1.05 and 1.10)) and maternal hospital admissions (relative risk of around 1.01) across all regions in Brazil (Zhao et al. 2019).

## Ambulance dispatches and emergency department presentations

Two studies (Kotani et al. 2018; Sun et al. 2014) investigated the impact of low-intensity heat events on all-cause ambulance dispatches (all ages). One found a positive association between a single-day heat event at lag 0 days, and all-cause ambulance dispatches with relative risks of 1.080 (1.050, 1.120) and temperatures ≥ 85^th^ percentile (Kotani et al. 2018). The other found a relative risk for all-cause ambulance dispatches of 1.060 (1.020, 1.101) for a single day with temperatures ≥ 90^th^ percentile, and 1.049 (1.014, 1.084) for a 2-day heatwave with temperatures ≥ 90^th^ percentile (Sun et al. 2014). One study (Yang et al. 2016a) investigated the impact of low-intensity heat events on ambulance dispatches for renal colic. There was a positive association between ambulance dispatches for renal colic and a single-day heat event ≥ 90^th^ percentile with a relative risk of 1.92 (1.21, 3.05).

Only one study investigated the association of low-intensity heat events and emergency department presentations (Sun et al. 2014). The impact of low-intensity heat events showed a small increase in risk, with a relative risk of emergency department presentations of 1.009 (0.992, 1.019) for single-day events, 1.026 (1.018, 1.035) for heat events of ≥ 2 days and 1.010 (1.002, 1.017) for ≥ 3 days with temperatures ≥ 90^th^ percentile.

### Discussion

Evidence collated here is inconsistent but suggestive that in regions with hot, humid summers, low-intensity heat events had a small but statistically significant impact on both morbidity and mortality. The level of risk varied between regions studied, with studies in some regions not showing an increase in morbidity and mortality during low-intensity heat events (here defined as 85–92.5^th^ percentile heat event) in regions with hot, humid summers. While the relative risk of mortality/morbidity was lower than during high-intensity heat events, a lower relative risk of mortality and morbidity can still be important at a population level. Our findings point to the importance of population-based and health system-related interventions to reduce the health impact of low-intensity heat events, with particular attention to the elderly, young children and those with chronic conditions. Our findings also suggest that, even in warm climates where populations and infrastructure are adapted to heat, there is a need for additional heat adaptation measures, particularly in the context of a warming climate (Tong et al. 2015).

While the evidence on the impact of extreme heat events on mortality is strongly suggestive, as shown in the systematic review by Xu et al. (2016), our findings also suggest that, in some climatic regions low-intensity heatwaves may also increase mortality. In their review, the authors (Xu et al. 2016) found that although there was a strong correlation between increasing heatwave intensity and increasing mortality risk, there was also evidence of an association between lower intensity events and mortality in some locations. Our review is unique in its focus on low-intensity heatwaves in regions with hot, humid summers.

In addition to an increase in all-cause mortality in most studies, our findings also indicate an increase in mortality for specific conditions including cardiovascular disease, respiratory disease and diabetes. A global systematic review and meta-analysis looking specifically at cardiovascular and respiratory mortality and morbidity reported similar findings, indicating an increase in mortality for both conditions during heatwave events (Cheng et al. 2019). It should be noted that this review differed from ours as it included studies from varying climate regions, focused on heatwaves only, excluded single-day events, and included both low- and high-intensity heatwaves. A few studies found an increase in cause-specific mortality due to a range of causes. Additional research on the impact of low-intensity heatwaves on these specific conditions may be warranted, as positive findings would point to the need for additional interventions targeting people with these conditions during low-intensity heat events.

While there was more consistency between studies of the impact of low-intensity heatwaves on mortality, we found only a small overall increase in hospital admissions during low-intensity events and that the impacts differed widely between age groups, with children under 4 years (Zhao et al. 2019) and adults over 80 (Zhao et al. 2019) being at the highest risk. Other less studied outcomes of measure for morbidity were ambulance dispatches and emergency department presentations, both of which indicated an increased risk during low-intensity events, but with a metric differing from hospital admissions and hence a different type of health outcome. This increased risk was low for emergency department presentations (Sun et al. 2014) but was higher for ambulance dispatches (Kotani et al. 2018; Sun et al. 2014; Yang et al. 2016a; Zhan et al. 2018). While we found only one study investigating emergency department presentations during low-intensity heat events, there are a number of published studies that investigated the impact of mid- to high-intensity heat events on emergency department presentations. Four studies, all conducted in Queensland, Australia, found a significant increase in emergency department presentations during heatwaves of a range of intensities, with the lowest of these being ≥ 95^th^ percentile (Toloo et al. 2014; Tong et al. 2010; Wang et al. 2012; Xu et al. 2019a, 2019d). The significant impact on emergency department presentations seen in mid-high intensity events and the increased risk of emergency department presentations in the one study that investigated the impact of low-intensity events in our review demonstrate the need for further research investigating the impact of low-intensity heat events on emergency department presentations.

As with mortality, hospital admissions during low-intensity heat events increased for some specific conditions. These conditions include respiratory diseases, RHD, heat-related illness and renal conditions. Even though we found that cardiovascular-related mortality is increased during low-intensity heat events, cardiovascular-related hospital admissions did not increase across all studies. This finding may be because cardiovascular-related deaths often occur from acute events such as myocardial infarction and stroke and that death may occur either before medical care arrives, during transit to hospital, or while in the emergency department and therefore does not result in hospital admission. This explanation is supported by data from an Australian and New Zealand out-of-hospital cardiac arrest (OHCA) database which found that out of all OHCA in 2015 in which resuscitation was attempted, only 28% survived the immediate event (Beck et al. 2018). There is a need for more research into the impact of low-intensity heat events on ambulance dispatches and emergency department presentations in regions with hot, humid summers, as these are more likely to capture a more accurate picture of demand on emergency and hospital services compared to investigating hospital admissions and mortality.

We found that the impact of low-intensity heatwaves on mortality (and to a lesser extent, morbidity) can be long-lasting, with effects being evident up to 21–28 days after the event (Yang et al. 2015). This finding is important because a large number of locations with hot, humid summers experience long, hot summers, often with multiple non-consecutive single high heat days and multi-day heat events throughout (Chien et al. 2016). The impact of multiple non-consecutive high heat days may be further compounded by small diurnal temperature ranges in the tropics/subtropics, which allow little time for physiological recovery (Bernhardt 2019; Nissan et al. 2017). With the effects from heat events potentially lasting up to 3 or 4 weeks later, there is a chance of another heat event occurring during that time, meaning that the health effects are likely to be prolonged.

The health impacts from prolonged heat as a result of multiple non-consecutive low-intensity heat events are not addressed within the current literature; however, the concept of health impacts from non-consecutive high-intensity heat days over a shorter period of time has been investigated in a study conducted in Hong Kong (Ho et al. 2017). This study investigated heat events with a variety of definitions including at least three very hot days/nights within a 7-day period and at least 5 very hot days/nights within a 7-day period. The authors’ rationale behind this was to explore whether high-intensity heat over non-consecutive hot days and nights would also contribute to excess mortality in the way that consecutive hot day and/or nights do. This paper was not included in our review as the definitions for the heatwave intensity thresholds as percentiles were unclear; however, they found that along with consecutive hot days/nights, non-consecutive hot days and nights also contribute significantly to mortality risk (Ho et al. 2017). This should be explored further, particularly for low-intensity heat events.

## Limitations and future research

Our findings highlight the limitations of the available evidence on the impact of low-intensity heat events on mortality and morbidity in regions that experience hot, humid summers. There are few papers that explicitly describe risk, and the differences in methods, definitions and control thresholds make comparison difficult. There were also a limited number of countries/regions studied with most studies focused on either lower-middle or upper middle-income countries, with little data available for high-income countries. Furthermore, some of the included publications provided relative risks of the impact of low-intensity events in graphical form only, due to their main focus being on high-intensity events. In these instances, relative risks were described as a range.

Unlike a systematic review where a clear research question is required a priori, we decided to undertake this review as a scoping review. We took this route as it allowed us to more extensively review the literature, without having a single research question in mind allowing several factors to be considered simultaneously. While the search strategy was finalised prior to undertaking the review, our inclusion and exclusion criteria were not. This approach may impact the replicability of the research and also have potentially introduced bias into which literature were included and excluded.

The majority of papers identified in this review found a statistically significant positive association between low-intensity heat events and morbidity and mortality. This may reflect publication bias with some research findings remaining unpublished. As we only searched for papers published in English, we may have missed relevant papers in other languages, particularly those describing findings of studies conducted in regions with hot, humid summers.

Further research into the impacts of low-intensity heat events on mortality is needed, particularly in high income countries, and regions with long, hot and humid summers. It is important to understand the point at which significant health impacts are likely to occur in these regions, to ensure that the appropriate heatwave response plans are made with the aim of reducing mortality and morbidity and reducing the strain on health systems.

There are also a limited number of studies on emergency department presentations which is an important outcome to consider when looking at impacts on hospital systems during low-intensity heat events. Further research looking at this particular outcome is recommended to provide a clearer indication on the effects that low-intensity heat events are likely to have on hospital systems. Additionally, future research into the effects of ambulance dispatches during low-intensity heat events in a wider range of locations should be conducted, as the four studies that investigated this outcome were all conducted in Asia.

### Conclusions

In this scoping review, we searched three databases for papers describing studies of the impact of low-intensity heat events on morbidity and mortality in regions with hot, humid summers. Our findings suggest that low-intensity heatwaves increase mortality in regions with hot, humid summers, but not to the same extent as high-intensity heatwaves do. The impact of low-intensity heatwaves on morbidity is uncertain, and there are few studies investigating its impact on emergency department visits and ambulance dispatches. For all health-related impacts of low-intensity heatwaves in regions with hot, humid summers, the evidence is less clear than for high-intensity heatwaves, and comparisons between studies are difficult. The mixed results point to the need for further research investigating these impacts, particularly in high-income countries.

## Supplementary Information

Below is the link to the electronic supplementary material.

Supplementary file1 (DOCX 18 KB)

Supplementary file2 (DOCX 131 KB)

## Data Availability

The dataset used for this study is available as supplementary material.
