# Supplementary material for: Impact of low-intensity heat events on mortality and morbidity in regions with hot, humid summers: a scoping literature review
Source: Int J Biometeorol. 2022 Jan 20;66(5):1013–29. doi: 10.1007/s00484-022-02243-z (PMC9042961; doi:10.1007/s00484-022-02243-z)
Supplement: Supplementary file 1 — Supplementary file1 (DOCX 18 KB) [file 484_2022_2243_MOESM1_ESM.docx]

**Table S1** Inclusion, exclusion criteria and search terms used in search strategy

| **PCC** | | **Inclusion** | **Exclusion** | **Search Terms** |
| --- | --- | --- | --- | --- |
| **Population:** | Humans | Papers that focus on how heat impacts humans directly | Papers focussing on how heat impacts animals  Papers that consider the impact of heat on humans via another organism (i.e. infection) | N/A |
| **Concept:** | Health impacts  associated with low intensity  heat events | Health impacts may include:  Mortality  Hospital admissions  ED presentations  Ambulance callouts  Papers that focus on low-intensity (92.5^th^ percentile and under) heat events | Paper only looks at high-intensity heat events (95^th^ percentile or over) or  does not clearly identify the level of intensity of the heat event studied  Papers that focus on severe weather events in general (do not specifically look at the impacts of heat alone) | morbidity OR "hospital admissions" OR "hospital presentations" OR ambulance OR "emergency department" OR "emergency admissions" OR "emergency presentations" OR mortality  AND  90th OR 92.5th OR 95th OR "low intensity" OR "excess heat factor"  AND  “prolonged heat” OR heatwave OR “heat index” OR humidex OR “extreme heat” OR “excess heat factor” OR “warm spells” OR "humidity"[MeSH Terms] OR "humidity"[All Fields] OR “Temperature/adverse effects"[Mesh] OR "Extreme Heat"[Mesh] OR “daily temperature*” OR “ambient temperature” |
| **Context:** | Tropical and humid- subtropical climates | Focus on climate that have a hot, humid summer  Focus on a range of climates but include at least one location with a hot, humid summer  Focus on middle/high income countries | Focus on climate classicisation other than humid sub-tropical or tropical  Focus on low income countries |  |
| **Other** |  |  | Systematic reviews (to avoid double ups, include relevant papers from the review)  Conference abstracts, editorials or other similar types of published literature  Studies not in English |  |
