# Supplementary material for: Impact of low-intensity heat events on mortality and morbidity in regions with hot, humid summers: a scoping literature review
Source: Int J Biometeorol. 2022 Jan 20;66(5):1013–29. doi: 10.1007/s00484-022-02243-z (PMC9042961; doi:10.1007/s00484-022-02243-z)
Supplement: Supplementary file 2 — Supplementary file2 (DOCX 131 KB) [file 484_2022_2243_MOESM2_ESM.docx]

**Table S2** Data collection table

Note: Data cited in the review from papers marked with an * have been converted from percent of increased risk to relative risk

| **Mortality (Non-accidental)** | | | | | | | | | | | |
| --- | --- | --- | --- | --- | --- | --- | --- | --- | --- | --- | --- |
| **Author (year)**  **(reference)** | **Location** | **Population** | **Years of Study** | **Heat Threshold** | **Control Threshold** | **Statistical analysis** | **Confounders Measured** | **Notes** | **Number of Health Events** | **Lags / Ages** | **Outcome Measure** |
| Ban  (2017) | China  (43 counties)  >1 Koppen classification; 38 counties with hot, humid summers | Not specified | 2013 - 2015 | Single day  90^th^ | 75^th^ | Quasi-Poisson regression model and distributed lag nonlinear model  - natural cubic spline 7df (time) 3df (relative humidity) | Air pollution (PM_2.5_ and O_3_)  Relative humidity  Control for seasonality and day of week | 0.32% missing data in total and cause-specific mortality 0.015% missing data in daily temperature. | 531,794 | 0-2  0-3  0-7 | RR  1.046 (1.034, 1.057)  1.037 (1.024, 1.051)  1.033 (1.016, 1.050) |
| Heo  (2019) | South Korea  (7 cities + 9 provinces)  >1 Koppen classification. Predominantly Dwa | 50.7 million | 2011 – 2014  Warm season (June - Sep) | AT 90^th^  2 days  Air Temp (1)  HI (2)  WBGT (3) | Non-heatwave day | Generalized additive model with a link function and a Quasi-Poisson distribution and piecewise regressions and distributed lag nonlinear model – 4df (lags) 2df (time and humidity) | Sensitivity analysis 3df for time | None | 308, 002  (total) | 0-20 (distributed)  0-1 (moving average) | RR  (1) 1.075 (1.027, 1.125) *single day*  (1) 0.964 (0.935–0.994)  *heat wave effect*  (2) 1.015 (0.999, 1.032) *single day*  (2) 1.033 (1.006, 1.061) *heat wave effect*  (3) 1.057 (1.012, 1.091) *single day*  (3) 1.014 (0.998–1.031) *heat wave effect*  (1) 1.051 (1.021, 1.082) *singe day*  (1) 0.970 (0.944–0.997) *heat wave effect*  (2) 1.007 (0.997, 1.016) *single day*  (2) 1.038 (1.013–1.065) *heat wave effect*  (3) 1.035 (1.005, 1.066) *single day*  (3) 1.021 (1.006–1.036) *heat wave effect* |
| Huang (2018)  (66) | Thailand  (60 provinces)  Koppen classification Aw, Am, Af | Not specified | 1999 -2008  Hot season (March – June) | 90^th^ – 93^rd^  (low intensity)  (2, 3, 4 days – pooled) | Non-heatwave day | Quasi-Poisson generalized additive model – natural cubic spline 3df (time) + random-effects meta-analysis | Relative humidity  Control for seasonality and day of the week | None | 457.14 (average per day) | 0-1  0-21 | RR  1.113 (1.097, 1.130)  1.169 (1.131, 1.208) |
| *Kent  (2014) | United States  (Alabama)  Koppen classification Cfa | Not specified | 1990 – 2010  Warm season (May – Sep) | 85^th^  1 day (1)  90^th^  1 day (2)  2 days (3) | Non-heatwave day | Time stratified case-crossover – natural cubic spline 3df (temperature) | Control for seasonality and day of the week | None | 301,126 | 0  1  6 | IR%  (1) 1.2 (-0.1 to 2.6)  (2) 2.0 (0.3 to 3.8)  (3) 3.7 (1.1 to 6.3)  (3) 4.1 (1.5 to 7.0)  (3) -1.5 (-4.5 to 1.0) |
| Kim  (2015) | South Korea  (Seoul)  All-cause mortality (including accidental)  Koppen classification Dwa | 9,794,304  (2010) | 1992 – 2009  Warm season (May – Sep) | 1°C increase in temp – roughly 93^rd^ percentile  (93^rd^: 30.3  95^th^: 31) | 90^th^ (29.5°C) | Over dispersed Poisson generalized linear model | Air pollution (PM_10_ and O_3_)  Relative humidity Air pressure  Control for seasonality and day of the week | Sensitivity analysis – lags 1, 2, 0-2, threshold temp 93, 95, 99 percentiles | 271,633  (total)  98.67  (average per day) | 0 | RR (all cause)  1.03 (1.02–1.03) |
| Lee  (2016) | South Korea  >1 Koppen classification, predominantly Dwa | 10,195,318  (2012) | 1992 – 2012  Warm season (May – Sep) | AT  90^th^  2 days (1)  3 days (2)  4 days (3) | Non-heatwave day | Generalized additive model and piecewise linear regression model – natural cubic spline – 3df (temperature), 4df (lags)  Added heatwave effect - distributed lag non-linear model – 3df (time), 4df (lags) | Control for seasonality and day of the week | None | 88.2  (average per day) | 0-30  Effects up to:  21 (1)  17 (2)  16 (3) | RR  (1) 1.037 (0.972, 1.106)  (2) 1.047 (0.985, 1.114)  (3) 1.058 (1.002, 1.118) |
| Nori-Sarma  (2019) | India  (Mumbai)  Koppen classification Aw  (4 other cities are not in included climate regions) | 1.5 million  (≥35 years old) | 2000 - 2012 | 90^th^  2 days (1)  3 days (2)  4 days (3)  92.5^th^  2 days (4)  3 days (5)  4 days (6) | Non-heatwave day | Quasi-Poisson regression model | Time trends,  Seasonal and cyclical variations, Day of the week, Adjusted dew point temperature (via propensity score matching) | None | 216, 635 | 0 | RR  (1), (2), (3), (4), (5): significantly greater than 1 and less than 1.1  (6): significantly greater than 1.1 and less than 1.2 |
| *Seposo  (2017) | Philippines  (4 cities)  >1 Koppen classification, predominantly Am, Af | 6,729,358  (2010) | 2006 – 2010  Warm season (Dec – May) | 90^th^  2 days (1)  4 days (2)  7 days (3) | 75^th^ | Quasi-Poisson regression, to account for over-dispersion coupled with distributed lag non-linear model – 3df (relative humidity), 7df (time) | Relative humidity  Control for seasonality and day of the week | None | 2.31 per 100,000 / day | All ages  0-14  15-64  65+ | IR%  (1) 12.5 (4.7, 20.9)  (2) 13.5 (3.1, 25.1)  (3) 11.2 (0.7, 22.8)  (1) 2.8 (−8.9, 15.9)  (2) 3.9 (−9.70, 19.7)  (3) 2.7 (−9.40, 16.5)  (1) 10.8 (1.30, 21.3)  (2) 12.5 (1.60, 24.5)  (3) 11.3 (−0.30, 24.2)  (1) 19.4 (10.9, 28.7)  (2) 22.7 (11.4, 35.2)  (3) 14.5 (0.60, 30.4) |
| *Son  (2011) | Seoul, South Korea  Koppen classification Dwa | Not specified | 2000 - 2007 | Single day  90^th^ | 50^th^ | Over-dispersed Poisson generalize linear model - natural cubic splines 7df (time/year), 3df (temperature, lags), 4df (humidity) | Air pollution (PM_10_ and O_3_)  Humidity  Control for seasonality and day of the week | None | 272,040 | Lag 0 All  0-14  15-64  65-74  75+ | IR%  9.30 (6.47, 12.19)  1.77 (−12.26, 18.06)  5.24 (0.95, 9.71)  6.82 (1.42, 12.51)  16.16 (11.79, 20.69) |
| Tong  (2014) | Australia (Brisbane, Sydney)  Koppen classification Cfa | 2.2 million  4.6 million | 1988 – 2009  Hot season (Dec – Feb) | 90^th^  2 days  Brisbane (1)  Sydney (2) | Non-heatwave day | Poisson generalised additive model | Humidity | Missing data (≤1.3%) Observations recorded from the remaining weather stations were used to compute the daily average values for missing data | 24  (Brisbane - average per day)  62  (Sydney - average per day) | Lags  0-2  All  Male  0-74  75+  Female  0-74  75+ | RR  (1) 1.04 (1.01, 1.07)  (2) 1.04 (1.03, 1.06)  (1) 0.99 (0.93 to 1.04)  (2) 1.04 (1.00 to 1.08)  (1) 1.03 (0.97 to 1.08)  (2) 1.03 (1.00 to 1.07)  (1) 1.04 (0.96 to 1.11)  (2) 1.04 (0.99 to 1.09)  (1) 1.07 (1.02 to 1.12)  (2) 1.06 (1.03 to 1.09) |
| Tong  (2015) | Australia (Brisbane, Sydney)  Koppen classification Cfa | 2.2 million  4.6 million | 1988 – 2009  Warm season (Nov - Mar) | 2 days  Brisbane  90^th^ (1)  92.5^th^ (2)  Sydney  90^th^ (3)  92.5^th^ (4) | Non-heatwave day | Poisson generalised additive model – natural cubic splines 3df (humidity), 4df (day of season).  A linear function was used for year to control for long-term trend | Humidity  Control for seasonality and day of the week  Sensitivity analyses - df for humidity (3–6 df), and for day of the warm season (3–6 df) | None | 24  (Brisbane - average per day)  62  (Sydney - average per day) | 0-3 | RR  (1) 1.08 [1.05 - 1.11]  (2) 1.09 [1.06 - 1.13]  (3) 1.06 [1.04 - 1.08]  (4) 1.08 [1.06 - 1.11] |
| Yang  (2019) | China  (31 cities)  >1 Koppen classification; 23 cities with hot, humid summers | 259.9 million | 2007 -2013  Warm season (May – Sep) | 90^th^  2 days (1)  3 days (2)  4 days (3)  92.5^th^  2 days (4)  3 days (5)  4 days (6) | Non-heatwave day | Poisson generalized linear model and distributed lag non-linear model – natural cubic spline 4df (seasonality, lags), 3df (air pollution), 5df (humidity and atmospheric pressure | Air pollution  Relative humidity  Atmospheric pressure  Control for seasonality and day of the week  Sensitivity analyses - df (3 to 6) and lag of days (14 to 21 days) for air pressure and relative humidity. Df (5 to 10) | No missing data | 2,315 (average per day) | Lag 0  All ages  (5)  All ages  Lag 0  0-2  0-10  0-64  Lag 0  0-2  0-10  65-74  Lag 0  0-2  0-10  75+  Lag 0  0-2  0-10 | RR  (1) 1.030 (1.020, 1.050)  (2) 1.040 (1.025, 1.070)  (3) 1.048 (1.027, 1.075)  (4) 1.044 (1.026, 1.065)  (5) 1.060 (1.030, 1.089)  (6) 1.060 (1.030, 1.090)  1.060 (1.030, 1.089)  1.09 (1.05, 1.13)  1.10 (1.05, 1.15)  1.04 (1.00,1.07)  1.04 (1.00,1.09)  1.04 (0.99,1.09)  1.05 (1.02,1.08)  1.08 (1.05,1.11)  1.07(1.01,1.14)  1.08 (1.04,1.11)  1.12 (1.07,1.17)  1.17 (1.09,1.24) |
| Yin  (2018) | China  (272 cities)  >1 Koppen classification; 242 cities with hot, humid summers | Not specified | 2013 - 2015 | 90^th^  2 days (1)  3 days (2)  4 days (3)  92.5^th^  2 days (4)  3 days (5)  4 days (6) | Non-heatwave day | Quasi-Poisson generalized additive model and a distributed lag model – natural cubic B-spline 4df (lag, seasonality), 6df (temperature) | Air pollution (PM_2.5_ and O_3_)  Relative humidity  Control for seasonality and day of the week | None | 1,826,186 | Lag  0-10  All  5-64  65-74  75+ | RR  (1) 1.016 (0.098, 1.032)  (2) 1.005 (0.085, 1.025)  (3) 1.015 (0.098, 1.028)  (4) 1.016 (0.098, 1.032)  (5) 1.020 (1.001, 1.037)  (6) 1.024 (1.005, 1.043)  (1) 0.980 (0.950, 1.010)  (2) 0.975 (0.945, 1.005)  (3) 0.980 (0.950, 1.010)  (4) 0.995 (0.960, 1.025)  (5) 1.000 (0.970, 1.025)  (6) 1.000 (0.970, 1.025)  (1) 0.985 (0.995, 1.015)  (2) 0.985 (0.960, 1.010)  (3) 1.000 (0.975, 1.025)  (4) 0.975 (0.940, 1.010)  (5) 0.995 (0.960, 1.025)  (6) 0.995 (0.960, 1.030)  (1) 1.050 (1.025, 1.075)  (2) 1.025 (1.010, 1.055)  (3) 1.030 (1.015, 1.055)  (4) 1.035 (1.020, 1.060)  (5) 1.035 (1.020, 1.060)  (6) 1.035 (1.020, 1.060) |
|  |  |  |  |  |  |  |  |  |  |  |  |
| HI = Heat Index; WBGT = Wet-bulb Globe Temperature; AT = Apparent Temperature; MMT = Minimum Mortality Temperature; RR = Relative Risk (95^th^ %CI); IR% = Increased Risk (%) | | | | | | | | | | | |

| **Mortality – All Circulatory Disease (ICD-10 I00-I99)** | | | | | | | | | | | |
| --- | --- | --- | --- | --- | --- | --- | --- | --- | --- | --- | --- |
| **Author (year)**  **(reference)** | **Location** | **Population** | **Years of Study** | **Heat Threshold** | **Control Threshold** | **Statistical analysis** | **Confounders Measured** | **Notes** | **Number of Health Events** | **Lags / Ages** | **Outcome Measure** |
| Ban  (2017) | China  (43 counties)  >1 Koppen classification; 38 counties with hot, humid summers | Not specified | 2013 - 2015 | Single day  90^th^ | 75^th^ | Quasi-Poisson regression model and distributed lag nonlinear model  - natural cubic spline 7df (time) 3df (relative humidity) | Air pollution (PM_2.5_ and O_3_)  Relative humidity  Control for seasonality and day of week | 0.32% missing data in total and cause-specific mortality 0.015% missing data in daily temperature. | 531,794 (total) | 0-2 | RR  1.040 (1.035,1.050) |
| Heo  (2019) | South Korea  (7 cities + 9 provinces)  >1 Koppen classification, predominantly Dwa | 50.7 million | 2011 – 2014  Warm season (June - Sep) | 90^th^  2 days  Air Temp (1)  HI (2)  WBGT (3) | Non-heatwave day | Generalized additive model with a link function and a Quasi-Poisson distribution and piecewise regressions and distributed lag nonlinear model – 4df (lags) 2df (time and humidity) | Sensitivity analysis 3df for time | None | 308, 002  (total) | 0-20 (distributed)  0-1 (moving average) | RR  (1) 0.994 (0.965, 1.024)  (2) 1.083 (1.012, 1.158)  (3) 1.021 (0.973, 1.072)  (1) 1.000 (0.970, 1.032)  (2) 1.076 (1.012, 1.145)  (3) 1.032 (0.986, 1.080) |
| Huang (2018) | Thailand  (60 provinces)  Koppen classification Aw, Am, Af | Not specified | 1999 -2008  Hot season (March – June) | 90^th^ – 93^rd^  (low intensity)  (2, 3, 4 days – pooled) | Non-heatwave day | Quasi-Poisson generalized additive model – natural cubic spline 3df (time) + random-effects meta-analysis | Relative humidity  Control for seasonality and day of the week | None | 457.14 (average per day, total) | 0-1  0-21 | RR  1.079 (1.051, 1.108)  1.129 (1.070, 1.190) |
| Kim  (2015) | South Korea  (Seoul)  Koppen classification Dwa | 9,794,304  (2010) | 1992 – 2009  Warm season (May – Sep) | 1°C increase in temp – roughly 93^rd^ percentile  (93^rd^: 30.3  95^th^: 31) | 90^th^ (29.5°C) | Over dispersed Poisson generalized linear model | Air pollution (PM_10_ and O_3_)  Relative humidity Air pressure  Control for seasonality and day of the week | Sensitivity analysis – lags 1, 2, 0-2, threshold temp 93, 95, 99 percentiles | 271,633  (total)  98.67  (average per day)  (total) | 0 | RR  1.04 (1.03–1.04) |
| Lee  (2016) | South Korea  >1 Koppen classification, predominantly Dwa | 10,195,318  (2012) | 1992 – 2012  Warm season (May – Sep) | AT  90^th^  2 days (1)  3 days (2)  4 days (3) | Non-heatwave day | Generalized additive model and piecewise linear regression model – natural cubic spline – 3df (temperature), 4df (lags)  Added heatwave effect - distributed lag non-linear model – 3df (time), 4df (lags) | Control for seasonality and day of the week | None | 88.2  (average per day, total) | 0-30  Effects up to:  21 (1)  17 (2)  16 (3) | RR  (1) 1.069 (0.945, 1.209)  (2) 1.125 (1.003, 1.263)  (3) 1.147 (1.035, 1.270) |
| *Seposo  (2017) | Philippines  (4 cities)  >1 Koppen classification, predominantly Am, Af | 6,729,358  (2010) | 2006 – 2010  Warm season (Dec – May) | 90^th^  2 days (1)  4 days (2)  7 days (3) | 75^th^ | Quasi-Poisson regression, to account for over-dispersion coupled with distributed lag non-linear model – 3df (relative humidity), 7df (time) | Relative humidity  Control for seasonality and day of the week | None | 0.74 per 100,000 / day | unclear | IR%  (1) 17.9 (6.40–30.7)  (2) 18.6 (5.90–32.9)  (3) 15.3 (2.30–30.1) |
| *Son  (2011) | Seoul, South Korea  Koppen classification Dwa | Not specified | 2000 - 2007 | Single day  90^th^ | 50^th^ | Over-dispersed Poisson generalize linear model - natural cubic splines 7df (time/year), 3df (temperature, lags), 4df (humidity) | Air pollution (PM_10_ and O_3_)  Humidity  Control for seasonality and day of the week | None | 272,040 (total) | Lag 0  All  0-14  15-64  65-74  75+ | IR%  6.07 (1.24, 11.13)  2.29 (−19.6, 30.14)  −2.17 (−10.43, 6.84)  8.55 (−1.01, 19.02)  9.41 (2.18, 17.15) |
| Yang  (2015) | China  Xuhui – central region in Shanghai  Koppen classification Cfa | 1.13 million | 1981 - 2012 | Single day  90^th^ | 26°C  75^th^ = 24.2°C  90^th^ = 28.5°C | Generalized additive quasi-Poisson models and distributed lag non-linear models – natural cubic spline 5df (temperature, lags), 7df (time), 3df (humidity) | Relative humidity  Control for seasonality and day of the week  Sensitivity analysis – minimum and max temps in place of mean, df (time) 6-10, df (temperature) 3-6 | None | 5  (average per day) | 0-1  0-3  0-7  0-14  0-21  0-28 | RR  1.04 (1.03,1.06)  1.04 (1.02,1.06)  1.04 (1.02,1.06)  1.03 (1.01,1.05)  1.04 (1.01,1.06)  1.04 (1.02,1.06) |
| Yang  (2019) | China  (31 cities)  >1 Koppen classification; 23 cities with hot, humid summers | 259.9 million | 2007 -2013  Warm season (May – Sep) | 92.5^th^  3 days | Non-heatwave day | Poisson generalized linear model and distributed lag non-linear model – natural cubic spline 4df (seasonality, lags), 3df (air pollution), 5df (humidity and atmospheric pressure | Air pollution  Relative humidity  Atmospheric pressure  Control for seasonality and day of the week  Sensitivity analyses - df (3 to 6) and lag of days (14 to 21 days) for air pressure and relative humidity. Df (5 to 10) | No missing data | 2,315 (average per day, total) | 0  0-2  0-10 | RR  1.08 (1.05,1.12)  1.13 (1.08,1.19)  1.16 (1.08,1.25) |
| Yin  (2018) | China  (272 cities)  >1 Koppen classification; 242 cities with hot, humid summers | Not specified | 2013 - 2015 | 90^th^  2 days (1)  3 days (2)  4 days (3)  92.5^th^  2 days (4)  3 days (5)  4 days (6) | Non-heatwave day | Quasi-Poisson generalized additive model and a distributed lag model – natural cubic B-spline 4df (lag, seasonality), 6df (temperature) | Air pollution (PM_2.5_ and O_3_)  Relative humidity  Control for seasonality and day of the week | None | 1,826,186 (total) | 0-10 | RR  (1) 1.050 (1.020, 1.070)  (2) 1.030 (1.010, 1.060)  (3) 1.040 (1.015, 1.065)  (4) 1.040 (1.010, 1.070)  (5) 1.050 (1.020, 1.070)  (6) 1.040 (1.010, 1.070) |
| HI = Heat Index; WBGT = Wet-bulb Globe Temperature; AT = Apparent Temperature; MMT = Minimum Mortality Temperature; RR = Relative Risk (95^th^ %CI); IR% = Increased Risk (%) | | | | | | | | | | | |

| **Mortality – Coronary Heart Disease (ICD-10 I20-I25)** | | | | | | | | | | | |
| --- | --- | --- | --- | --- | --- | --- | --- | --- | --- | --- | --- |
| **Author (year)**  **(reference)** | **Location** | **Population** | **Years of Study** | **Heat Threshold** | **Control Threshold** | **Statistical analysis** | **Confounders Measured** | **Notes** | **Number of Health Events** | **Lags** | **Outcome Measure** |
| Ban  (2017) | China  (43 counties)  >1 Koppen classification; 38 counties with hot, humid summers | Not specified | 2013 - 2015 | Single day  90^th^ | 75^th^ | Quasi-Poisson regression model and distributed lag nonlinear model  - natural cubic spline 7df (time) 3df (relative humidity) | Air pollution (PM_2.5_ and O_3_)  Relative humidity  Control for seasonality and day of week | 0.32% missing data in total and cause-specific mortality 0.015% missing data in daily temperature. | 531,794 (total) | 0-2 | RR  1.015 (1.010,1.020) |
| Huang (2018) | Thailand  (60 provinces)  Koppen classification Aw, Am, Af | Not specified | 1999 -2008  Hot season (March – June) | 90^th^ – 93^rd^  (low intensity)  (2, 3, 4 days – pooled) | Non-heatwave day | Quasi-Poisson generalized additive model – natural cubic spline 3df (time) + random-effects meta-analysis | Relative humidity  Control for seasonality and day of the week | None | 457.14 (average per day, total) | 0-1  0-21 | RR  1.171 (1.116,1.229)  1.187 (1.082, 1.301) |
| Kim  (2015) | South Korea  (Seoul)  Koppen classification Dwa | 9,794,304  (2010) | 1992 – 2009  Warm season (May – Sep) | 1°C increase in temp – roughly 93^rd^ percentile  (93^rd^: 30.3  95^th^: 31) | 90^th^ (29.5°C) | Over dispersed Poisson generalized linear model | Air pollution (PM_10_ and O_3_)  Relative humidity Air pressure  Control for seasonality and day of the week | Sensitivity analysis – lags 1, 2, 0-2, threshold temp 93, 95, 99 percentiles | 271,633  (total)  98.67  (average per day)  (total) | 0 | RR  1.02 (1.00–1.04) |
| *Tian  (2013) | China  (Beijing)  Koppen classification Dwa | Not specified | 2000 – 2011  Hot season (May – Sep) | 87.5^th^  2 days (1)  3 days (2)  4 days (3)  90^th^  2 days (4)  3 days (5)  4 days (6)  92.5^th^  2 days (7)  3 days (8)  4 days (9) | Non-heatwave day | Quasi-Poisson regression model and distributed lag non-linear model – natural cubic spline 5df (humidity), 4df (lags and seasonality) | Relative humidity  Control for seasonality and day of the week  Sensitivity analyses – relative humidity df 3-6 and lag 10-30. Seasonality – df 5-10 | No missing data | 5  (average per day for CHD only) | Lag 0 -15  All ages  <65  65+ | IR%  (1) 13.7 (5.7, 21.6)  (2) 16.5 (7.7, 25.3)  (3) 15.7 (6.1, 25.4)  (4) 15.3 (6.4, 24.1)  (5) 18.3 (8.5, 28.1)  (6) 18.5 (7.1, 29.9)  (7) 18.1 (8.4, 27.8)  (8) 18.3 (7.9, 28.7)  (9) 16.2 (3.8, 28.7)  (1) 11.8 (2.7, 20.8)  (2) 13.8 (3.7, 23.9)  (3) 14.8 (3.8, 25.7)  (4) 10.8 (0.7, 21)  (5) 12.2 (0.9, 23.4)  (6) 13.0 (-0.2, 26.1)  (7) 12.2 (1.0, 23.4)  (8) 11.3 (-0.7, 23.3)  (9) 11.5 (-2.8, 25.8)  (1) 18.4 (3.7, 33.1)  (2) 23.2 (7.0, 39.5)  (3) 18.1 (0.1, 36.2)  (4) 26.4 (10.2, 42.5)  (5) 33.4 (15.7, 51.1)  (6) 32.2 (11.5, 52.8)  (7) 32.6 (15.0, 50.2)  (8) 35.4 (16.8, 54)  (9) 28.3 (5.7, 50.9) |
| Yang  (2019) | China  (31 cities)  >1 Koppen classification; 23 cities with hot, humid summers | 259.9 million | 2007 -2013  Warm season (May – Sep) | 92.5^th^  3 days | Non-heatwave day | Poisson generalized linear model and distributed lag non-linear model – natural cubic spline 4df (seasonality, lags), 3df (air pollution), 5df (humidity and atmospheric pressure | Air pollution  Relative humidity  Atmospheric pressure  Control for seasonality and day of the week  Sensitivity analyses - df (3 to 6) and lag of days (14 to 21 days) for air pressure and relative humidity. Df (5 to 10) | No missing data | 2,315 (average per day, total) | 0  0-2  0-10 | RR  1.09 (1.05,1.14)  1.14 (1.08,1.21)  1.21 (1.10,1.32) |
| Yin  (2018) | China  (272 cities)  >1 Koppen classification; 242 cities with hot, humid summers | Not specified | 2013 - 2015 | 90^th^  2 days (1)  3 days (2)  4 days (3)  92.5^th^  2 days (4)  3 days (5)  4 days (6) | Non-heatwave day | Quasi-Poisson generalized additive model and a distributed lag model – natural cubic B-spline 4df (lag, seasonality), 6df (temperature) | Air pollution (PM_2.5_ and O_3_)  Relative humidity  Control for seasonality and day of the week | None | 1,826,186 | 0-10 | RR  (1) 1.045 (1.010, 1.080)  (2) 1.030 (1.010, 1.060)  (3) 1.030 (1.000, 1.070)  (4) 1.030 (0.900, 1.075)  (5) 1.030 (1.000, 1.075)  (6) 1.030 (0.090, 1.060) |
| MMT = Minimum Mortality Temperature; RR = Relative Risk (95^th^ %CI); IR% = Increased Risk (%) | | | | | | | | | | | |

| **Mortality – Stroke (ICD-10 I60-I69 / I60-I64)** | | | | | | | | | | | |
| --- | --- | --- | --- | --- | --- | --- | --- | --- | --- | --- | --- |
| **Author (year)**  **(reference)** | **Location** | **Population** | **Years of Study** | **Heat Threshold** | **Control Threshold** | **Statistical analysis** | **Confounders Measured** | **Notes** | **Number of Health Events** | **Lags** | **Outcome Measure** |
| Ban  (2017) | China  (43 counties)  >1 Koppen classification; 38 counties with hot, humid summers | Not specified | 2013 - 2015 | Single day  90^th^ | 75^th^ | Quasi-Poisson regression model and distributed lag nonlinear model  - natural cubic spline 7df (time) 3df (relative humidity) | Air pollution (PM_2.5_ and O_3_)  Relative humidity  Control for seasonality and day of week | 0.32% missing data in total and cause-specific mortality 0.015% missing data in daily temperature. | 531,794 (total) | 0-2 | RR  1.020 (1.010,1.030) |
| Chen  (2013) | China  (8 cities), all but one (Taiyuan, Bsk) had included Koppen classifications | 47.7 million | 1996 - 2008 | Single day  90^th^ | 75^th^ | Quasi-Poisson generalized additive model  and Bayesian hierarchical model – natural cubic spline 7df (time), 5df (temperature), 3df (confounders), 4df (lags) | Air pollution (PM_10_, SO_2_ and NO_2_)  Relative humidity  Control for seasonality and day of the week  Sensitivity analysis: AT | No missing data | 97  (average per day, stroke only) | 0-3  0-7  0-14  0-21  0-28 | RR  1.06 (1.02–1.10)  1.04 (0.96–1.12)  1.05 (0.98–1.12)  1.02 (0.93–1.13)  1.03 (0.89–1.18) |
| Kim  (2015) | South Korea  (Seoul)  Koppen classification Dwa | 9,794,304  (2010) | 1992 – 2009  Warm season (May – Sep) | 1°C increase in temp – roughly 93^rd^ percentile  (93^rd^: 30.3  95^th^: 31) | 90^th^ (29.5°C)  Over dispersed Poisson generalized linear model | Over dispersed Poisson generalized linear model | Air pollution (PM_10_ and O_3_)  Relative humidity Air pressure  Control for seasonality and day of the week | Sensitivity analysis – lags 1, 2, 0-2, threshold temp 93, 95, 99 percentiles | 271,633  (total)  98.67  (average per day)  (total) | 0 | RR  1.04 (1.03–1.06) |
| Yang  (2019) | China  (31 cities)  >1 Koppen classification; 23 cities with hot, humid summers | 259.9 million | 2007 -2013  Warm season (May – Sep) | 92.5^th^  3 days | Non-heatwave day | Poisson generalized linear model and distributed lag non-linear model – natural cubic spline 4df (seasonality, lags), 3df (air pollution), 5df (humidity and atmospheric pressure | Air pollution  Relative humidity  Atmospheric pressure  Control for seasonality and day of the week  Sensitivity analyses - df (3 to 6) and lag of days (14 to 21 days) for air pressure and relative humidity. Df (5 to 10) | No missing data | 2,315 (average per day, total) | 0  0-2  0-10 | RR  1.08 (1.04,1.11)  1.13 (1.07,1.19)  1.17 (1.08,1.27) |
| Yin  (2018) | China  (272 cities)  >1 Koppen classification; 242 cities with hot, humid summers | Not specified | 2013 - 2015 | 90^th^  2 days (1)  3 days (2)  4 days (3)  92.5^th^  2 days (4)  3 days (5)  4 days (6) | Non-heatwave day | Quasi-Poisson generalized additive model and a distributed lag model – natural cubic B-spline 4df (lag, seasonality), 6df (temperature) | Air pollution (PM_2.5_ and O_3_)  Relative humidity  Control for seasonality and day of the week | None | 1,826,186 | 0-10 | RR  (1) 1.040 (1.010, 1.070)  (2) 1.020 (0.090, 1.045)  (3) 1.030 (1.000, 1.055)  (4) 1.030 (1.000, 1.060)  (5) 1.040 (1.005, 1.070)  (6) 1.050 (1.020, 1.080) |
| MMT = Minimum Mortality Temperature; RR = Relative Risk (95^th^ %CI); IR% = Increased Risk (%) | | | | | | | | | | | |

| **Mortality – Respiratory Disease (ICD-10 J00-J99 / J30-J98)** | | | | | | | | | | | |
| --- | --- | --- | --- | --- | --- | --- | --- | --- | --- | --- | --- |
| **Author (year)**  **(reference)** | **Location** | **Population** | **Years of Study** | **Heat Threshold** | **Control Threshold** | **Statistical analysis** | **Confounders Measured** | **Notes** | **Number of Health Events** | **Lags** | **Outcome Measure** |
| Ban  (2017) | China  (43 counties)  >1 Koppen classification; 38 counties with hot, humid summers | Not specified | 2013 - 2015 | Single day  90^th^ | 75^th^ | Quasi-Poisson regression model and distributed lag nonlinear model  - natural cubic spline 7df (time) 3df (relative humidity) | Air pollution (PM_2.5_ and O_3_)  Relative humidity  Control for seasonality and day of week | 0.32% missing data in total and cause-specific mortality 0.015% missing data in daily temperature. | 531,794 (total) | 0-2 | RR  1.050 (1.035,1.060) |
| Heo  (2019) | South Korea  (7 cities + 9 provinces)  >1 Koppen classification, predominantly Dwa | 50.7 million | 2011 – 2014  Warm season (June - Sep) | 90^th^  2 days  Air Temp (1)  HI (2)  WBGT (3) | Non-heatwave day | Generalized additive model with a link function and a Quasi-Poisson distribution and piecewise regressions and distributed lag nonlinear model – 4df (lags) 2df (time and humidity) | Sensitivity analysis 3df for time | None | 308, 002  (total) | 0-20 (distributed)  0-1 (moving average) | RR  (1) 1.065 (0.932–1.216)  (2) 1.056 (0.921–1.211)  (3) 1.094 (0.949–1.262)  (1) 1.032 (0.929–1.148)  (2) 1.040 (0.910–1.189)  (3) 1.075 (0.941–1.229) |
| Huang (2018) | Thailand  (60 provinces)  Koppen classification Aw, Am, Af | Not specified | 1999 -2008  Hot season (March – June) | 90^th^ – 93^rd^  (low intensity)  (2, 3, 4 days – pooled) | Non-heatwave day | Quasi-Poisson generalized additive model – natural cubic spline 3df (time) + random-effects meta-analysis | Relative humidity  Control for seasonality and day of the week | None | 457.14 (average per day, total) | 0-1  0-21 | RR  1.105 (1.068, 1.143)  1.145 (1.081, 1.212) |
| Kim  (2015) | South Korea  (Seoul)  Koppen classification Dwa | 9,794,304  (2010) | 1992 – 2009  Warm season (May – Sep) | 1°C increase in temp – roughly 93^rd^ percentile  (93^rd^: 30.3  95^th^: 31) | 90^th^ (29.5°C) | Over dispersed Poisson generalized linear model | Air pollution (PM_10_ and O_3_)  Relative humidity Air pressure  Control for seasonality and day of the week | Sensitivity analysis – lags 1, 2, 0-2, threshold temp 93, 95, 99 percentiles | 271,633  (total)  98.67  (average per day)  (total) | 0 | RR  1.02 (1.00–1.04) |
| Lee  (2016) | South Korea  >1 Koppen classification, predominantly Dwa | 10,195,318  (2012) | 1992 – 2012  Warm season (May – Sep) | AT  90^th^  2 days (1)  3 days (2)  4 days (3) | Non-heatwave day | Generalized additive model and piecewise linear regression model – natural cubic spline – 3df (temperature), 4df (lags)  Added heatwave effect - distributed lag non-linear model – 3df (time), 4df (lags) | Control for seasonality and day of the week | None | 88.2  (average per day, total) | 0-30  Effects up to:  21 (1)  17 (2)  16 (3) | RR  (1) 1.057 (0.778, 1.436)  (2) 1.080 (0.806, 1.448)  (3) 1.129 (0.871, 1.464) |
| *Seposo  (2017) | Philippines  (4 cities)  >1 Koppen classification, predominantly Am, Af | 6,729,358  (2010) | 2006 – 2010  Warm season (Dec – May) | 90^th^  2 days (1)  4 days (2)  7 days (3) | 75^th^ | Quasi-Poisson regression, to account for over-dispersion coupled with distributed lag non-linear model – 3df (relative humidity), 7df (time) | Relative humidity  Control for seasonality and day of the week | None | 0.255 per 100,000 / day | unclear | IR%  (1) 31.5 (14.1–51.5)  (2) 37.3 (16.2–62.3)  (3) 23.2 (6.30–42.8) |
| *Son  (2011) | Seoul, South Korea  Koppen classification Dwa | Not specified | 2000 - 2007 | Single day  90^th^ | 50^th^ | Over-dispersed Poisson generalize linear model - natural cubic splines 7df (time/year), 3df (temperature, lags), 4df (humidity) | Air pollution (PM_10_ and O_3_)  Humidity  Control for seasonality and day of the week | None | 272,040 (total) | Lag 0 All  0-14  15-64  65-74  75+ | IR%  12.01 (1.25, 23.92)  6.3 (−18.45, 38.57)  4.59 (−11.15, 23.12)  3.53 (–11.21, 20.71)  6.12 (−6.08, 19.91) |
| Yang  (2019) | China  (31 cities)  >1 Koppen classification; 23 cities with hot, humid summers | 259.9 million | 2007 -2013  Warm season (May – Sep) | 92.5^th^  3 days | Non-heatwave day | Poisson generalized linear model and distributed lag non-linear model – natural cubic spline 4df (seasonality, lags), 3df (air pollution), 5df (humidity and atmospheric pressure | Air pollution  Relative humidity  Atmospheric pressure  Control for seasonality and day of the week  Sensitivity analyses - df (3 to 6) and lag of days (14 to 21 days) for air pressure and relative humidity. Df (5 to 10) | No missing data | 2,315 (average per day, total) | 0  0-2  0-10 | RR  1.06 (1.03,1.10)  1.12 (1.06,1.18)  1.17 (1.07,1.28) |
| Yin  (2018) | China  (272 cities)  >1 Koppen classification; 242 cities with hot, humid summers | Not specified | 2013 - 2015 | 90^th^  2 days (1)  3 days (2)  4 days (3)  92.5^th^  2 days (4)  3 days (5)  4 days (6) | Non-heatwave day | Quasi-Poisson generalized additive model and a distributed lag model – natural cubic B-spline 4df (lag, seasonality), 6df (temperature) | Air pollution (PM_2.5_ and O_3_)  Relative humidity  Control for seasonality and day of the week | None | 1,826,186 | 0-10 | RR  (1) 1.060 (1.020, 1.100)  (2) 1.050 (1.005, 1.090)  (3) 1.030 (0.090, 1.070)  (4) 1.065 (1.015, 1.115)  (5) 1.050 (1.000, 1.095)  (6) 1.050 (1.000, 1.090) |
| HI = Heat Index; WBGT = Wet-bulb Globe Temperature; AT = Apparent Temperature; RR = Relative Risk (95^th^ %CI); IR% = Increased Risk (%) | | | | | | | | | | | |

| **Mortality – Diabetes (ICD-10 E10-E14)** | | | | | | | | | | | |
| --- | --- | --- | --- | --- | --- | --- | --- | --- | --- | --- | --- |
| **Author (year)**  **(reference)** | **Location** | **Population** | **Years of Study** | **Heat Threshold** | **Control Threshold** | **Statistical analysis** | **Confounders Measured** | **Notes** | **Number of Health Events** | **Lags** | **Outcome Measure** |
| He  (2020) | Thailand  (60 provinces)  Koppen classification Aw, Am, Af | Not specified | 2000 – 2008 | Single day  90^th^ | MMT (location specific) | Time-stratified case-crossover analysis (distributed lag model) – natural cubic spline 5df (temperature), 3df (confounders) and conditional logistic regression | Relative Humidity  Diurnal temperature  Control for seasonality and day of week  Sensitivity analysis – 75^th^ percentile as control | 16 provinces were omitted due to missing data | 59, 836 | 0  0-1  0-2  0-3  0-4  0-5  0-6 | OR  1.10 (1.06–1.15)  1.10 (1.06–1.14)  1.06 (1.03–1.10)  1.05 (1.01–1.09)  1.00 (1.00–1.01)  1.01 (1.00–1.02)  1.00 (1.00–1.00) |
| Huang  (2018) | Thailand  (60 provinces)  Koppen classification Aw, Am, Af | Not specified | 1999 -2008  Hot season (March – June) | 90^th^ – 93^rd^  (low intensity)  (2, 3, 4 days – pooled) | Non-heatwave day | Quasi-Poisson generalized additive model – natural cubic spline 3df (time) + random-effects meta-analysis | Relative humidity  Control for seasonality and day of the week | None | 457.14 (average per day, total) | 0-1  0-21 | RR  1.125 (1.070, 1.182)  1.260 (1.141, 1.390) |
| Yang  (2016b) | China  (9 cities)  All but one city (Kunming) were in regions with included Koppen classification | 129.3 million | 2007-2013 | Single day  90^th^ | 75^th^ | Distributed lag non-linear model combined with quasi-Poisson regression – natural cubic spline 7df (time), 3df (confounders), 5df (lags, temperature) | Relative humidity  Barometric pressure  Control for seasonality and day of the week  Sensitivity analysis - df (6–11 per year) for time, df (3–6) for confounders, lag 20 to 30. | No missing data | 30 (average per day, diabetes only) | Lag  0-21  All  0-75  75+ | RR  1.11 (1.03,1.19)  1.07 (0.96,1.20)  1.16 (1.04,1.28) |
| Yang  (2019) | China  (31 cities)  >1 Koppen classification; 23 cities with hot, humid summers | 259.9 million | 2007 -2013  Warm season (May – Sep) | 92.5^th^  3 days | Non-heatwave day | Poisson generalized linear model and distributed lag non-linear model – natural cubic spline 4df (seasonality, lags), 3df (air pollution), 5df (humidity and atmospheric pressure | Air pollution  Relative humidity  Atmospheric pressure  Control for seasonality and day of the week  Sensitivity analyses - df (3 to 6) and lag of days (14 to 21 days) for air pressure and relative humidity. Df (5 to 10) | No missing data | 2,315 (average per day, total) | 0  0-2  0-10 | RR  1.08 (1.00,1.17)  1.15 (1.06,1.26)  1.16 (1.03,1.31) |
| MMT = Minimum Mortality Temperature; RR = Relative Risk (95^th^ %CI); OR = Odds Ratio | | | | | | | | | | | |

| **Mortality – Other** | | | | | | | | | | | |
| --- | --- | --- | --- | --- | --- | --- | --- | --- | --- | --- | --- |
| **Author (year)**  **(reference)** | **Location** | **Population / Condition** | **Years of Study** | **Heat Threshold** | **Control Threshold** | **Statistical analysis** | **Confounders Measured** | **Notes** | **Number of Health Events** | **Lags** | **Outcome Measure** |
| Ban  (2017) | China  (43 counties)  >1 Koppen classification; 38 counties with hot, humid summers | Not specified  Acute MI | 2013 - 2015 | Single day  90^th^ | 75^th^ | Quasi-Poisson regression model and distributed lag nonlinear model  - natural cubic spline 7df (time) 3df (relative humidity) | Air pollution (PM_2.5_ and O_3_)  Relative humidity  Control for seasonality and day of week | 0.32% missing data in total and cause-specific mortality 0.015% missing data in daily temperature. | 531,794 (total) | 0-2 | RR  1.040 (1.020,1.055) |
| Kim  (2015) | South Korea  (Seoul)  Koppen classification Dwa | 9,794,304  (2010)  MI  I20-I23 | 1992 – 2009  Warm season (May – Sep) | 1°C increase in temp – roughly 93^rd^ percentile  (93^rd^: 30.3  95^th^: 31) | 90^th^ (29.5°C) | Over dispersed Poisson generalized linear model | Air pollution (PM_10_ and O_3_)  Relative humidity Air pressure  Control for seasonality and day of the week | Sensitivity analysis – lags 1, 2, 0-2, threshold temp 93, 95, 99 percentiles | 271,633  (total)  98.67  (average per day)  (total) | 0 | RR  1.02 (1.00–1.04) |
| Kim  (2015) | South Korea  (Seoul)  Koppen classification Dwa | 9,794,304  (2010)  Hypertensive disease  I10-I15 | 1992 – 2009  Warm season (May – Sep) | 1°C increase in temp – roughly 93^rd^ percentile  (93^rd^: 30.3  95^th^: 31) | 90^th^ (29.5°C) | Over dispersed Poisson generalized linear model | Air pollution (PM_10_ and O_3_)  Relative humidity Air pressure  Control for seasonality and day of the week | Sensitivity analysis – lags 1, 2, 0-2, threshold temp 93, 95, 99 percentiles | 271,633  (total)  98.67  (average per day)  (total) | 0 | RR  1.04 (1.00–1.07) |
| Kim  (2015) | South Korea  (Seoul)  Koppen classification Dwa | 9,794,304  (2010)  Heart Failure  I50 | 1992 – 2009  Warm season (May – Sep) | 1°C increase in temp – roughly 93^rd^ percentile  (93^rd^: 30.3  95^th^: 31) | 90^th^ (29.5°C) | Over dispersed Poisson generalized linear model | Air pollution (PM_10_ and O_3_)  Relative humidity Air pressure  Control for seasonality and day of the week | Sensitivity analysis – lags 1, 2, 0-2, threshold temp 93, 95, 99 percentiles | 271,633  (total)  98.67  (average per day)  (total) | 0 | RR  1.01 (0.97–1.06) |
| Kim  (2015) | South Korea  (Seoul)  Koppen classification Dwa | 9,794,304  (2010)  Chronic Ischemic Heart Disease  I05-I09 | 1992 – 2009  Warm season (May – Sep) | 1°C increase in temp – roughly 93^rd^ percentile  (93^rd^: 30.3  95^th^: 31) | 90^th^ (29.5°C) | Over dispersed Poisson generalized linear model | Air pollution (PM_10_ and O_3_)  Relative humidity Air pressure  Control for seasonality and day of the week | Sensitivity analysis – lags 1, 2, 0-2, threshold temp 93, 95, 99 percentiles | 271,633  (total)  98.67  (average per day)  (total) | 0 | RR  1.01 (0.96–1.07) |
| Kim  (2015) | South Korea  (Seoul)  Koppen classification Dwa | 9,794,304  (2010)  Asthma  J45-J46 | 1992 – 2009  Warm season (May – Sep) | 1°C increase in temp – roughly 93^rd^ percentile  (93^rd^: 30.3  95^th^: 31) | 90^th^ (29.5°C) | Over dispersed Poisson generalized linear model | Air pollution (PM_10_ and O_3_)  Relative humidity Air pressure  Control for seasonality and day of the week | Sensitivity analysis – lags 1, 2, 0-2, threshold temp 93, 95, 99 percentiles | 271,633  (total)  98.67  (average per day)  (total) | 0 | RR  1.05 (1.01–1.10) |
| Kim  (2015) | South Korea  (Seoul)  Koppen classification Dwa | 9,794,304  (2010)  COPD  J40-J44 | 1992 – 2009  Warm season (May – Sep) | 1°C increase in temp – roughly 93^rd^ percentile  (93^rd^: 30.3  95^th^: 31) | 90^th^ (29.5°C) | Over dispersed Poisson generalized linear model | Air pollution (PM_10_ and O_3_)  Relative humidity Air pressure  Control for seasonality and day of the week | Sensitivity analysis – lags 1, 2, 0-2, threshold temp 93, 95, 99 percentiles | 271,633  (total)  98.67  (average per day)  (total) | 0 | RR  1.01 (0.97–1.04) |
| Yang  (2019) | China  (31 cities)  >1 Koppen classification; 23 cities with hot, humid summers | 259.9 million  COPD | 2007 -2013  Warm season (May – Sep) | 92.5^th^  3 days | Non-heatwave day |  | Air pollution | None | 2,315 (average per day, total) | 0  0-2  0-10 | RR  1.04 (1.00,1.09)  1.08 (1.03,1.14)  1.13 (1.05,1.21) |
| Yin  (2018) | China  (272 cities)  >1 Koppen classification; 242 cities with hot, humid summers | Not specified  COPD | 2013 - 2015 | 90^th^  2 days (1)  3 days (2)  4 days (3)  92.5^th^  2 days (4)  3 days (5)  4 days (6) | Non-heatwave day | Quasi-Poisson generalized additive model and a distributed lag model – natural cubic B-spline 4df (lag, seasonality), 6df (temperature) | Air pollution (PM_2.5_ and O_3_)  Relative humidity  Control for seasonality and day of the week | None | 1,826,186 | 0-10 | RR  (1) 1.045 (1.005, 1.090)  (2) 1.045 (1.000, 1.095)  (3) 1.020 (0.070, 1.070)  (4) 1.040 (0.080, 1.100)  (5) 1.030 (0.075, 1.080)  (6) 1.025 (0.070, 1.075) |
| Huang  (2018) | Thailand  (60 provinces)  Koppen classification Aw, Am, Af | Not specified  Infectious / Parasitic disease  A00-B99 | 1999 -2008  Hot season (March – June) | 90^th^ – 93^rd^  (low intensity)  (2, 3, 4 days – pooled) | Non-heatwave day | Quasi-Poisson generalized additive model – natural cubic spline 3df (time) + random-effects meta-analysis | Relative humidity  Control for seasonality and day of the week | None | 457.14 (average per day, total) | 0-1  0-21 | RR  1.183 (1.146, 1.221)  1.268 (1.175, 1.369) |
| Huang (2018) | Thailand  (60 provinces) Koppen classification Aw, Am, Af | Not specified  Pneumonia  J12-J18 | 1999 -2008  Hot season (March – June) | 90^th^ – 93^rd^  (low intensity)  (2, 3, 4 days – pooled) | Non-heatwave day | Quasi-Poisson generalized additive model – natural cubic spline 3df (time) + random-effects meta-analysis | Relative humidity  Control for seasonality and day of the week | None | 457.14 (average per day, total) | 0-1  0-21 | RR  1.150 (1.096, 1.207)  1.155 (1.052, 1.267) |
| Kim  (2015) | South Korea  (Seoul)  Koppen classification Dwa | 9,794,304  (2010)  Pneumonia  J09-J22 | 1992 – 2009  Warm season (May – Sep) | 1°C increase in temp – roughly 93^rd^ percentile  (93^rd^: 30.3  95^th^: 31) | 90^th^ (29.5°C) | Over dispersed Poisson generalized linear model | Air pollution (PM_10_ and O_3_)  Relative humidity Air pressure  Control for seasonality and day of the week | Sensitivity analysis – lags 1, 2, 0-2, threshold temp 93, 95, 99 percentiles | 271,633  (total)  98.67  (average per day)  (total) | 0 | RR  1.03 (0.99–1.07) |
| Huang (2018) | Thailand  (60 provinces)  Koppen classification Aw, Am, Af | Not specified  Malignant Neoplasms  C00- C97 | 1999 -2008  Hot season (March – June) | 90^th^ – 93^rd^  (low intensity)  (2, 3, 4 days – pooled) | Non-heatwave day | Quasi-Poisson generalized additive model – natural cubic spline 3df (time) + random-effects meta-analysis | Relative humidity  Control for seasonality and day of the week | None | 457.14 (average per day, total) | 0-1  0-21 | RR  1.139 (1.101, 1.179)  1.211 (1.118, 1.312) |
| Huang (2018) | Thailand  (60 provinces)  Koppen classification Aw, Am, Af | Not specified  Neoplasms  C00-D48 | 1999 -2008  Hot season (March – June) | 90^th^ – 93^rd^  (low intensity)  (2, 3, 4 days – pooled) | Non-heatwave day | Quasi-Poisson generalized additive model – natural cubic spline 3df (time) + random-effects meta-analysis | Relative humidity  Control for seasonality and day of the week | None | 457.14 (average per day, total) | 0-1  0-21 | RR  1.139 (1.100,1.179)  1.222 (1.132, 1.320) |
| Huang (2018) | Thailand  (60 provinces)  Koppen classification Aw, Am, Af | Not specified  Digestive system disease  K00-K93 | 1999 -2008  Hot season (March – June) | 90^th^ – 93^rd^  (low intensity)  (2, 3, 4 days – pooled) | Non-heatwave day | Quasi-Poisson generalized additive model – natural cubic spline 3df (time) + random-effects meta-analysis | Relative humidity  Control for seasonality and day of the week | None | 457.14 (average per day, total) | 0-1  0-21 | RR  1.132 (1.080, 1.186)  1.175 (1.087, 1.270) |
| Kim  (2015) | South Korea  (Seoul)  Koppen classification Dwa | 9,794,304  (2010)  Digestive system disease  K00-K93 | 1992 – 2009  Warm season (May – Sep) | 1°C increase in temp – roughly 93^rd^ percentile  (93^rd^: 30.3  95^th^: 31) | 90^th^ (29.5°C) | Over dispersed Poisson generalized linear model | Air pollution (PM_10_ and O_3_)  Relative humidity Air pressure  Control for seasonality and day of the week | Sensitivity analysis – lags 1, 2, 0-2, threshold temp 93, 95, 99 percentiles | 271,633  (total)  98.67  (average per day)  (total) | 0 | RR  1.01 (0.99–1.03) |
| Huang (2018) | Thailand  (60 provinces)  Koppen classification Aw, Am, Af | Not specified  Endocrine, nutritional and metabolic diseases  E00-E90 | 1999 -2008  Hot season (March – June) | 90^th^ – 93^rd^  (low intensity)  (2, 3, 4 days – pooled) | Non-heatwave day | Quasi-Poisson generalized additive model – natural cubic spline 3df (time) + random-effects meta-analysis | Relative humidity  Control for seasonality and day of the week | None | 457.14 (average per day, total) | 0-1  0-21 | RR  1.127 (1.077, 1.180)  1.220 (1.108, 1.344) |
| Kim  (2015) | South Korea  (Seoul)  Koppen classification Dwa | 9,794,304  (2010)  Endocrine, nutritional and metabolic diseases  E00-E99 | 1992 – 2009  Warm season (May – Sep) | 1°C increase in temp – roughly 93^rd^ percentile  (93^rd^: 30.3  95^th^: 31) | 90^th^ (29.5°C) | Over dispersed Poisson generalized linear model | Air pollution (PM_10_ and O_3_)  Relative humidity Air pressure  Control for seasonality and day of the week | Sensitivity analysis – lags 1, 2, 0-2, threshold temp 93, 95, 99 percentiles | 271,633  (total)  98.67  (average per day)  (total) | 0 | RR  1.03 (1.01–1.05) |
| Huang (2018) | Thailand  (60 provinces)  Koppen classification Aw, Am, Af | Not specified  Renal failure  N17-19 | 1999 -2008  Hot season (March – June) | 90^th^ – 93^rd^  (low intensity)  (2, 3, 4 days – pooled) | Non-heatwave day | Quasi-Poisson generalized additive model – natural cubic spline 3df (time) + random-effects meta-analysis | Relative humidity  Control for seasonality and day of the week | None | 457.14 (average per day, total) | 0-1  0-21 | RR  1.037 (0.975, 1.103)  1.010 (0.910, 1.121) |
| Huang (2018) | Thailand  (60 provinces)  Koppen classification Aw, Am, Af | Not specified  Diseases of the genitourinary system  N00-N99 | 1999 -2008  Hot season (March – June) | 90^th^ – 93^rd^  (low intensity)  (2, 3, 4 days – pooled) | Non-heatwave day | Quasi-Poisson generalized additive model – natural cubic spline 3df (time) + random-effects meta-analysis | Relative humidity  Control for seasonality and day of the week | None | 457.14 (average per day, total) | 0-1  0-21 | RR  1.036 (0.981, 1.095)  1.012 (0.937, 1.092) |
| Kim  (2015) | South Korea  (Seoul)  Koppen classification Dwa | 9,794,304  (2010)  Diseases of the genitourinary system  N00-N99 | 1992 – 2009  Warm season (May – Sep) | 1°C increase in temp – roughly 93^rd^ percentile  (93^rd^: 30.3  95^th^: 31) | 90^th^ (29.5°C) | Over dispersed Poisson generalized linear model | Air pollution (PM_10_ and O_3_)  Relative humidity Air pressure  Control for seasonality and day of the week | Sensitivity analysis – lags 1, 2, 0-2, threshold temp 93, 95, 99 percentiles | 271,633  (total)  98.67  (average per day)  (total) | 0 | RR  1.05 (1.02–1.09) |
| Kim  (2015) | South Korea  (Seoul)  Koppen classification Dwa | 9,794,304  (2010)  All accidents / injury  S, T, V01-Y98 | 1992 – 2009  Warm season (May – Sep) | 1°C increase in temp – roughly 93^rd^ percentile  (93^rd^: 30.3  95^th^: 31) | 90^th^ (29.5°C) | Over dispersed Poisson generalized linear model | Air pollution (PM_10_ and O_3_)  Relative humidity Air pressure  Control for seasonality and day of the week | Sensitivity analysis – lags 1, 2, 0-2, threshold temp 93, 95, 99 percentiles | 271,633  (total)  98.67  (average per day)  (total) | 0 | RR  1.03 (1.02–1.04) |
| Kim  (2015) | South Korea  (Seoul)  Koppen classification Dwa | 9,794,304  (2010)  Sudden Death  I46 | 1992 – 2009  Warm season (May – Sep) | 1°C increase in temp – roughly 93^rd^ percentile  (93^rd^: 30.3  95^th^: 31) | 90^th^ (29.5°C) | Over dispersed Poisson generalized linear model | Air pollution (PM_10_ and O_3_)  Relative humidity Air pressure  Control for seasonality and day of the week | Sensitivity analysis – lags 1, 2, 0-2, threshold temp 93, 95, 99 percentiles | 271,633  (total)  98.67  (average per day)  (total) | 0 | RR  1.04 (1.01–1.08) |
| Kim  (2015) | South Korea  (Seoul)  Koppen classification Dwa | 9,794,304  (2010)  Nervous System Disease  G00-G99 | 1992 – 2009  Warm season (May – Sep) | 1°C increase in temp – roughly 93^rd^ percentile  (93^rd^: 30.3  95^th^: 31) | 90^th^ (29.5°C) | Over dispersed Poisson generalized linear model | Air pollution (PM_10_ and O_3_)  Relative humidity Air pressure  Control for seasonality and day of the week | Sensitivity analysis – lags 1, 2, 0-2, threshold temp 93, 95, 99 percentiles | 271,633  (total)  98.67  (average per day)  (total) | 0 | RR  1.07 (1.04–1.11) |
| Kim  (2015) | South Korea  (Seoul)  Koppen classification Dwa | 9,794,304  (2010)  Blood and Immune Mechanism Disease  D50-D89 | 1992 – 2009  Warm season (May – Sep) | 1°C increase in temp – roughly 93^rd^ percentile  (93^rd^: 30.3  95^th^: 31) | 90^th^ (29.5°C) | Over dispersed Poisson generalized linear model | Air pollution (PM_10_ and O_3_)  Relative humidity Air pressure  Control for seasonality and day of the week | Sensitivity analysis – lags 1, 2, 0-2, threshold temp 93, 95, 99 percentiles | 271,633  (total)  98.67  (average per day)  (total) | 0 | RR  1.02 (0.93–1.11) |
| Kim  (2015) | South Korea  (Seoul)  Koppen classification Dwa | 9,794,304  (2010)  Mental and Behavioural Disorders  F00-F99 | 1992 – 2009  Warm season (May – Sep) | 1°C increase in temp – roughly 93^rd^ percentile  (93^rd^: 30.3  95^th^: 31) | 90^th^ (29.5°C) | Over dispersed Poisson generalized linear model | Air pollution (PM_10_ and O_3_)  Relative humidity Air pressure  Control for seasonality and day of the week | Sensitivity analysis – lags 1, 2, 0-2, threshold temp 93, 95, 99 percentiles | 271,633  (total)  98.67  (average per day)  (total) | 0  All  Organic  Substance use  Schizophre-nia  Self-harm | RR  1.04 (1.01–1.07)  1.03 (0.99–1.07)  1.07 (1.02–1.13)  1.00 (0.86–1.15)  0.97 (0.95–0.99) |
| RR = Relative Risk (95^th^ %CI) | | | | | | | | | | | |

| **All-Cause Morbidity – Hospital Admissions** | | | | | | | | | | | |
| --- | --- | --- | --- | --- | --- | --- | --- | --- | --- | --- | --- |
| **Author (year)**  **(reference)** | **Location** | **Population** | **Years of Study** | **Heat Threshold** | **Control Threshold** | **Statistical analysis** | **Confounders Measured** | **Notes** | **Number of Health Events** | **Lags** | **Outcome Measure** |
| *Gronlund  (2014) | United States  (114 cities)  >1 Koppen classification. City-only analysis for New York and Houston (both Cfa) | Not Specified  Elderly (≥ 65 years) | 1992-2006  Warm season (May – Sep) | AT  Single Day  90^th^ | 75^th^ | Time stratified case crossover – natural cubic spline 3 df (temperature) | Air pollution (O_3_)  Sensitivity analysis conducted using mean, minimum, maximum and diurnal temperature range  Control for day and seasonality | Cities were excluded if they were missing data for more than 15% of study days. | 186 (average per day, all cause, all cities) | 0-1  0-7 | Small but significant increased risk for New York city at lag 0, larger significant increased risk for New York city at lags 0-7. No significant increase for Houston. |
| Xu  (2017) | Australia  (Brisbane)  Koppen classification Cfa | Not Specified  Infants (<1 year) | 2005 - 2015 | 90^th^  ≥2 days (1)  ≥3 days (2)  ≥4 days (3) | Non-heatwave day | Poisson generalized additive model allowing for overdispersion.  Distributed lag non-linear model to capture lagged effects – 7 df (time). Other dfs - based on the minimum generalized cross validation  Case only | Air pollution  (PM_10_ and NO_2_)  Relative humidity  Control for seasonality  Sensitivity analyses - df (time) 7 - 10 | None | 53,792  (total) | Not Specifi-ed | RR  (1) 1.01 (0.90, 1.04)  (2) 1.01 (0.70, 1.04)  (3) 1.01 (0.70, 1.06) |
| *Zhao  (2019) | Brazil  (1814 cities)  >1 Koppen classification; Af, Am, Aw, Cfa, Csa, Cfb, Cwb (71% of cases), and As, BsH (29% of cases) | Not Specified | 2000 – 2015  5 hottest months each year (city specific) | 90^th^  ≥2 days (1)  ≥3 days (2)  ≥4 days (3)  92.5^th^  ≥2 days (4)  ≥3 days (5)  ≥4 days (6) | Non-heatwave day | Stage 1: quasi-Poisson regression with constrained distributed lag model – 3 degrees of freedom (lag)  Stage 2: city-specific estimates were pooled at the national level using a random-effect meta-analysis with maximum likelihood estimation | Relative humidity  Control for seasonality  Sensitivity analyses - lag from 0–7 days to 0–9 days and df of lag days from three to five. | The 1814 cities were chosen as they had complete hospital data for the study period | 58,400,682  (total) | Lag0-7  All ages  Ages 0-4  Ages 5-9  Ages 10-19  Ages 20-29  Ages 30-39  Ages 40-49  Ages 50-59  Ages 60-69  Ages 70-79  Ages 80+ | IR%  Small (<2.5%) significant increased risk nationally for 90^th^ and 92.5^th^ percentiles and heatwaves of all durations.  Small (4 - 6%) significant increased risk nationally for 90^th^ and 92.5^th^ percentiles and heatwaves of all durations.  Small (3 - 5%) significant increased risk nationally for 90^th^ and 92.5^th^ percentiles and heatwaves of all durations.  Small (<2.5%) significant increased risk nationally for 90^th^ and 92.5^th^ percentiles and heatwaves of all durations.  Small (<2.5%) significant increased risk nationally for 90^th^ and 92.5^th^ percentiles and heatwaves of all durations.  Small (<1.25%) significant increased risk nationally for 90^th^ and 92.5^th^ percentiles and heatwaves of all durations except for 90^th^ percentile ≥4 days.  No significant increased risk nationally for 90^th^ and 92.5^th^ percentiles and heatwaves of all durations.  No significant increased risk nationally for 90^th^ and 92.5^th^ percentiles and heatwaves of all durations.  No significant increased risk nationally for 90^th^ and 92.5^th^ percentiles and heatwaves of all durations except for 92.5^th^ percentile ≥4 days.  Significant (4 - 8%) increased risk nationally for 90^th^ and 92.5^th^ percentiles and heatwaves of all durations.  Significant (4 - 8%) increased risk nationally for 90^th^ and 92.5^th^ percentiles and heatwaves of all durations. |
| **All-Cause Morbidity – Emergency Department Visits** | | | | | | | | | | | |
| *Sun  (2014) | China  (Pudong New Area, Shanghai)  Koppen classification Cfa | 5.26 million | Jan 2011 – Aug 2013  Warm season (May – Sep) | 90^th^  1 day (1)  ≥2 days (2)  ≥3 days (3) | Non-heatwave day | Over-dispersed Poisson generalized additive model - degrees of freedom for trend was selected automatically by the Generalized Cross Validation criterion | Relative humidity  Control for day and seasonality  Sensitivity analysis conducted using minimum and maximum temperature and a range of df (9-17) | None | 4,450,556  (total)  4,579 (average per day) | 0-7 | IR%  (1) 0.92 (−0.08, 1.93)  (2) 2.62 (1.78, 3.46)  (3) 0.95 (0.22, 1.69) |
| **All-Cause Morbidity – Ambulance Dispatches** | | | | | | | | | | | |
| Kotani  (2018) | Japan  (Fukuoka)  Koppen classification Cfa | 1.46 million | 2005 – 2012  Warm season (May – Sep) | Single day  85^th^ (1)  95^th^ (2) | MMT  (age group specific) | Quasi-Poisson regression using the distributed lag nonlinear model – natural cubic spline 4 df (temperature, confounders and lag), 5 df (time) | Air pollution (PM_2.5_)  Relative humidity  Control for day and seasonality | None | 107,041  87.5  (average per day, all-cause) | Lag 0  All Ages  Ages 0-19  Ages 20-39  Ages 40-59  Ages 60-79  Ages 80+ | RR  (1) 1.08 (1.05, 1.12)  (2) 1.12 (1.08, 1.16)  (1) 1.10 (1.00, 1.20)  (2) 1.11 (1.00, 1.23)  (1) 1.13 (1.05, 1.20)  (2) 1.17 (1.10, 1.26)  (1) 1.11 (1.04, 1.18)  (2) 1.15 (1.06, 1.23)  (1) 1.04 (0.98, 1.10)  (2) 1.06 (1.02, 1.13)  (1) 1.08 (1.02, 1.16)  (2) 1.10 (1.02, 1.19) |
| *Sun  (2014) | China  (Pudong New Area, Shanghai)  Koppen classification Cfa | 5.26 million | Jan 2011 – Aug 2013  Warm season (May – Sep) | 90^th^  1 day (1)  ≥2 days (2)  ≥3 days (3) | Non-heatwave day | Over-dispersed Poisson generalized additive model - degrees of freedom for trend was selected automatically by the Generalized Cross Validation criterion | Relative humidity  Control for day and seasonality  Sensitivity analysis conducted using minimum and maximum temperature and a range of df (9-17) | None | 246,372  (total)  253 (average per day) | 0-7 | IR%  (1) 6.03 (2.03, 10.18)  (2) 4.85 (1.42, 8.39)  (3) 3.94 (0.88, 7.10) |
| AT = Apparent Temperature; MMT = Minimum Mortality Temperature; RR = Relative Risk (95^th^ %CI); IR% = Increased Risk (%) | | | | | | | | | | | |

| **Cardiovascular Morbidity - Hospital Admissions** | | | | | | | | | | | |
| --- | --- | --- | --- | --- | --- | --- | --- | --- | --- | --- | --- |
| **Author (year)**  **(reference)** | **Location** | **Population** | **Years of Study** | **Heat Threshold** | **Control Threshold** | **Statistical analysis** | **Confounders Measured** | **Notes** | **Number of Health Events** | **Lags** | **Outcome Measure** |
| Cui  (2019) | China  (Hefei)  7 hospitals (more than 50% of CVD hospitalisations)  Koppen classification Cfa | 7.87 million | July 2015 – October 2017 | Single day  90^th^ | 75^th^ | Distributed-lag nonlinear model – 7 df (time), 3 degrees of freedom (weather variable confounders). 5 df (temperature) | Air pollution  (PM_10_, SO_2_ and NO_2_)  Relative humidity  Control for day and seasonality  Sensitivity analysis - changing the df for time (6, 8, 9) per year and altering df (2,4,5) for relative humidity and air pollutants | None | 35,096  (CVD only)  42  (average per day, CVD only) | All ages  Lags:  0  0-3  0-7  0-14  0-21  0-27  <65 yrs  ≥65 yrs  Lag  0-27(?) | RR  1.015 (0.988–1.043)  0.970 (0.876–1.075)  0.996 (0.958–1.036)  0.980 (0.907–1.059)  0.961 (0.852–1.085)  0.943 (0.805–1.107)  0.982 (0.933–1.035)  1.081 (1.012–1.154) |
| Ge  (2018) | China  (Shanghai)  Koppen classification Cfa | 24 million  Rheumatic Heat Disease  (I05-I09) | 2013 - 2015 | Single Day  90^th^ | 0°C | Quasi-Poisson generalized additive model and distributed lag nonlinear model – 3 df (temperature, humidity and lags), 7 df (time) | Air pollution (PM_2.5_ and O_3_)  Relative humidity  Control for day and seasonality  Sensitivity analysis conducted for air pollution, df of time to 8 and humidity to 6 | No missing data for temperature, humidity and air pollution | 3.8  (average per day, RHD only) | All ages  Lags:  0-2  0-3  0-4  0-5  <65 yrs  Lags:  0-2  0-3  0-4  0-5  ≥65 yrs  Lags:  0-2  0-3  0-4  0-5 | RR  2.62 (1.40–4.91)  2.58 (1.29–5.15)  2.66 (1.25–5.67)  2.70 (1.19–6.15)  1.25 (0.54–2.87)  1.16 (0.46–2.93)  1.69 (0.60–4.71)  2.60 (0.82–8.18)  4.55 (2.00–10.33)  4.13 (1.66–10.26)  3.57 (1.33–9.57)  3.04 (1.05–8.83) |
| *Gronlund  (2014) | United States  (114 cities)  >1 Koppen classification. City-only analysis not available for cause-specific morbidity | Not Specified  Elderly (≥ 65 years) | 1992-2006  Warm season (May – Sep) | AT  Single Day  90^th^ | 75^th^ | Time stratified case crossover – natural cubic spline 3 degrees of freedom | Air pollution (O_3_)  Sensitivity analysis conducted using mean, minimum, maximum and diurnal temperature range | Cities were excluded if they were missing data for more than 15% of study days. | 45 (average per day, CVD only) | 0-1  0-7 | IR%  –0.4 (–0.6, –0.2)  –1.3 (–1.6, –1.0) |
| Heo  (2019) | South Korea  (7 cities + 9 provinces)  >1 Koppen classification, predominantly Dwa | 50.7 million | 2011 – 2014  Warm season (June - Sep) | 90^th^  2 days  Air Temp (1)  HI (2)  WBGT (3) | Non-heatwave day | Generalized additive model with a link function and a Quasi-Poisson distribution and piecewise regressions and distributed lag nonlinear model – 4df (lags) 2df (time and humidity) | Sensitivity analysis 3df for time | None | Not specified | 0-20 (distributed)  0-1 (moving average) | RR  (1) 1.025 (0.929–1.130)  (2) 0.973 (0.894–1.059)  (3) 1.010 (0.932–1.096)  (1) 1.036 (0.952–1.128)  (2) 1.001 (0.933–1.074)  (3) 1.013 (0.963–1.066) |
| *Zhao  (2019) | Brazil  (1814 cities)  >1 Koppen classification; Af, Am, Aw, Cfa, Csa, Cfb, Cwb (71% of cases), and As, BsH (29% of cases) | Not Specified | 2000 – 2015  5 hottest months each year (city specific) | 90^th^  ≥2 days (1)  ≥3 days (2)  ≥4 days (3)  92.5^th^  ≥2 days (4)  ≥3 days (5)  ≥4 days (6) | Non-heatwave day  See Zhao all-cause morbidity for statistical analysis | Stage 1: quasi-Poisson regression with constrained distributed lag model – 3 degrees of freedom (lag)  Stage 2: city-specific estimates were pooled at the national level using a random-effect meta-analysis with maximum likelihood estimation | Relative humidity  Control for seasonality  Sensitivity analyses - lag from 0–7 days to 0–9 days and df of lag days from three to five. | The 1814 cities were chosen as they had complete hospital data for the study period | 58,400,682  (total) | 0-7 | Small (<2.5%) significant risk decrease across 90^th^ and 92.5^th^ percentiles and all heatwave durations |
| HI = Heat Index; WBGT = Wet-bulb Globe Temperature; AT = Apparent Temperature; RR = Relative Risk (95^th^ %CI); IR% = Increased Risk (%) | | | | | | | | | | | |

| **Respiratory Morbidity - Hospital Admissions** | | | | | | | | | | | |
| --- | --- | --- | --- | --- | --- | --- | --- | --- | --- | --- | --- |
| **Author (year)**  **(reference)** | **Location** | **Population** | **Years of Study** | **Heat Threshold** | **Control Threshold** | **Statistical analysis** | **Confounders Measured** | **Notes** | **Number of Health Events** | **Lags** | **Outcome Measure** |
| Heo  (2019) | South Korea  (7 cities + 9 provinces)  >1 Koppen classification, predominantly Dwa | 50.7 million | 2011 – 2014  Warm season (June - Sep) | 90^th^  2 days  Air Temp (1)  HI (2)  WBGT (3) | Non-heatwave day | Generalized additive model with a link function and a Quasi-Poisson distribution and piecewise regressions and distributed lag nonlinear model – 4df (lags) 2df (time and humidity) | Sensitivity analysis 3df for time | None | Not specified | 0-20 (distributed)  0-1 (moving average) | RR  (1) 0.983 (0.917–1.053)  (2) 1.011 (0.942–1.086)  (3) 1.027 (0.972–1.084)  (1) 1.055 (0.985–1.131)  (2) 1.131 (1.028–1.245)  (3) 1.124 (1.038–1.217) |
| *Gronlund  (2014) | United States  (114 cities)  >1 Koppen classification. City-only analysis not available for cause-specific morbidity | Not Specified  Elderly (≥ 65 years) | 1992-2006  Warm season (May – Sep) | AT  Single Day  90^th^ | 75^th^ | Time stratified case crossover – natural cubic spline 3 degrees of freedom | Air pollution (O_3_)  Sensitivity analysis conducted using mean, minimum, maximum and diurnal temperature range | Cities were excluded if they were missing data for more than 15% of study days. | 16 (average per day, respiratory only) | 0-1  0-7 | IR%  1.3 (0.8, 1.8)  0.0 (–0.6, 0.7) |
| Zhang  (2020) | China  (Beijing) | 22 million  COPD (ICD-10: J41–J44) | 2013 - 2016 | Mean temp (1)  AT (2)  Single Day  90^th^ | 75^th^ | Quasi-Poisson function combined with a distributed lag non-linear model – natural cubic spline 7df (time), 3df (confounders), 4df (temperature) | Relative humidity Wind speed  Air pressure  Air quality index  Control for seasonality and day of week | No missing data | 143, 318  (total) | Lag  0-30  All ages  <65 yrs  ≥65 yrs | RR  (1) 1.09(0.93,1.26)  (2) 1.07(0.92,1.24)  (1) 1.03 (0.82,1.30)  (2) 1.01 (0.81,1.27)  (1) 1.10 (0.94,1.28)  (2) 1.08 (0.93,1.26) |
| *Zhao  (2019) | Brazil  (1814 cities)  >1 Koppen classification; Af, Am, Aw, Cfa, Csa, Cfb, Cwb (71% of cases), and As, BsH (29% of cases) | Not Specified | 2000 – 2015  5 hottest months each year (city specific) | 90^th^  ≥2 days (1)  ≥3 days (2)  ≥4 days (3)  92.5^th^  ≥2 days (4)  ≥3 days (5)  ≥4 days (6) | Non-heatwave day | Stage 1: quasi-Poisson regression with constrained distributed lag model – 3 degrees of freedom (lag)  Stage 2: city-specific estimates were pooled at the national level using a random-effect meta-analysis with maximum likelihood estimation | Relative humidity  Control for seasonality  Sensitivity analyses - lag from 0–7 days to 0–9 days and df of lag days from three to five. | The 1814 cities were chosen as they had complete hospital data for the study period | 58,400,682  (total) | 0-7 | Small (<5%) significant risk increase across 90^th^ and 92.5^th^ percentiles and heatwave durations |
| HI = Heat Index; WBGT = Wet-bulb Globe Temperature; AT = Apparent Temperature; RR = Relative Risk (95^th^ %CI); IR% = Increased Risk (%) | | | | | | | | | | | |

| **Renal Morbidity - Hospital Admissions** | | | | | | | | | | | |
| --- | --- | --- | --- | --- | --- | --- | --- | --- | --- | --- | --- |
| **Author (year)**  **(reference)** | **Location** | **Population** | **Years of Study** | **Heat Threshold** | **Control Threshold +** | **Statistical analysis** | **Confounders Measured** | **Notes** | **Number of Health Events** | **Lags** | **Outcome Measure** |
| *Gronlund  (2014) | United States  (114 cities)  >1 Koppen classification. City-only analysis not available for cause-specific morbidity | Not Specified  Elderly (≥ 65 years) | 1992-2006  Warm season (May – Sep) | AT  Single Day  90^th^ | 75^th^ | Time stratified case crossover – natural cubic spline 3 degrees of freedom | Air pollution (O_3_)  Sensitivity analysis conducted using mean, minimum, maximum and diurnal temperature range | Cities were excluded if they were missing data for more than 15% of study days. | 2.2 (average per day, renal only) | 0-1  0-7 | IR%  3.9 (2.9, 4.9)  4.3 (3.0, 5.6) |
| AT = Apparent Temperature; IR% = Increased Risk (%) | | | | | | | | | | | |

| **Renal Colic – Ambulance Dispatches** | | | | | | | | | | | |
| --- | --- | --- | --- | --- | --- | --- | --- | --- | --- | --- | --- |
| **Author (year)**  **(reference)** | **Location** | **Population** | **Years of Study** | **Heat Threshold** | **Control Threshold** | **Statistical analysis** | **Confounders Measured** | **Notes** | **Number of Health Events** | **Lags** | **Outcome Measure** |
| Yang  (2016a) | China  (Guangzhou) | 12.78 million | 2008 - 2012 | 90^th^  Single Day | Mean temperature (21°C) | Distributed-lag nonlinear model and over-dispersed generalized additive model  – natural cubic splines 3df (temperature, lags), 7df (time) | Relative humidity  Control for seasonality and day of the week  Sensitivity analysis – minimum and maximum temperature, df (time) 4-10 | No missing data | 3158  (total) | 0-7 | RR  1.92 (1.21, 3.05) |
| RR = Relative Risk (95^th^ %CI) | | | | | | | | | | | |

| **Genito-urinary Morbidity - Hospital Admissions** | | | | | | | | | | | |
| --- | --- | --- | --- | --- | --- | --- | --- | --- | --- | --- | --- |
| **Author (year)**  **(reference)** | **Location** | **Population** | **Years of Study** | **Heat Threshold** | **Control Threshold +** | **Statistical analysis** | **Confounders Measured** | **Notes** | **Number of Health Events** | **Lags** | **Outcome Measure** |
| *Zhao  (2019) | Brazil  (1814 cities)  >1 Koppen classification; Af, Am, Aw, Cfa, Csa, Cfb, Cwb (71% of cases), and As, BsH (29% of cases) | Not Specified | 2000 – 2015  5 hottest months each year (city specific) | 90^th^  ≥2 days (1)  ≥3 days (2)  ≥4 days (3)  92.5^th^  ≥2 days (4)  ≥3 days (5)  ≥4 days (6) | Non-heatwave day | Stage 1: quasi-Poisson regression with constrained distributed lag model – 3 degrees of freedom (lag)  Stage 2: city-specific estimates were pooled at the national level using a random-effect meta-analysis with maximum likelihood estimation | Relative humidity  Control for seasonality  Sensitivity analyses - lag from 0–7 days to 0–9 days and df of lag days from three to five. | The 1814 cities were chosen as they had complete hospital data for the study period | 58,400,682  (total) | 0-7 | Small (<5%) significant risk increase across 90^th^ and 92.5^th^ percentiles and heatwave durations |
| IR% = Increased Risk (%) | | | | | | | | | | | |

| **Endocrine, Nutritional, Metabolic Morbidity - Hospital Admissions** | | | | | | | | | | | |
| --- | --- | --- | --- | --- | --- | --- | --- | --- | --- | --- | --- |
| **Author (year)**  **(reference)** | **Location** | **Population** | **Years of Study** | **Heat Threshold** | **Control Threshold** | **Statistical analysis** | **Confounders Measured** | **Notes** | **Number of Health Events** | **Lags** | **Outcome Measure** |
| *Zhao  (2019) | Brazil  (1814 cities)  >1 Koppen classification; Af, Am, Aw, Cfa, Csa, Cfb, Cwb (71% of cases), and As, BsH (29% of cases) | Not Specified | 2000 – 2015  5 hottest months each year (city specific) | 90^th^  ≥2 days (1)  ≥3 days (2)  ≥4 days (3)  92.5^th^  ≥2 days (4)  ≥3 days (5)  ≥4 days (6) | Non-heatwave day | Stage 1: quasi-Poisson regression with constrained distributed lag model – 3 degrees of freedom (lag)  Stage 2: city-specific estimates were pooled at the national level using a random-effect meta-analysis with maximum likelihood estimation | Relative humidity  Control for seasonality  Sensitivity analyses - lag from 0–7 days to 0–9 days and df of lag days from three to five. | The 1814 cities were chosen as they had complete hospital data for the study period | 58,400,682  (total) | 0-7 | Significant risk increase (5 – 10%) across 90^th^ and 92.5^th^ percentiles and heatwave durations; higher in 92.5^th^ percentile than 90^th^ percentile |
| IR% = Increased Risk (%) | | | | | | | | | | | |

| **Diabetes Morbidity - Hospital Admissions and Post-Discharge Deaths** | | | | | | | | | | | |
| --- | --- | --- | --- | --- | --- | --- | --- | --- | --- | --- | --- |
| **Author (year)**  **(reference)** | **Location** | **Population** | **Years of Study** | **Heat Threshold** | **Control Threshold** | **Statistical analysis** | **Confounders Measured** | **Notes** | **Number of Health Events** | **Lags** | **Outcome Measure** |
| Xu  (2019b) | Australia  (Brisbane) | Not specified | 2005 – 2013  Post-discharge deaths up to 2 months after discharge | 90^th^  ≥2 days | Non-heatwave day | Time-stratified case-crossover design with conditional logistic regression and case-only design with binary or multinomial logistic regression (individual- and community level characteristics) | Air pollution  (PM_10_ and NO_2_)  Relative humidity  Confounders controlled using natural cubic spline (3df) | None | 10,542  (total)  513 (post-discharge deaths) | All ages  Lags:  0  1  2  0-14  ≥65 yrs  All ages  Lags  0  1  2  0-14  ≥65 yrs | OR  1.09 (0.97, 1.23)  1.04 (0.93, 1.18)  0.97 (0.86, 1.10)  1.36 (1.04, 1.78)  0.96 (0.83, 1.11)  1.15 (0.82, 1.61)  1.26 (0.90, 1.78)  1.46 (1.03, 2.07)  N/A  0.55 (0.23, 1.10) |
| OR = Odds Ratio (95% CI) | | | | | | | | | | | |

| **Neoplasm Morbidity - Hospital Admissions** | | | | | | | | | | | |
| --- | --- | --- | --- | --- | --- | --- | --- | --- | --- | --- | --- |
| **Author (year)**  **(reference)** | **Location** | **Population** | **Years of Study** | **Heat Threshold** | **Control Threshold** | **Statistical analysis** | **Confounders Measured** | **Notes** | **Number of Health Events** | **Lags** | **Outcome Measure** |
| *Zhao  (2019) | Brazil  (1814 cities)  >1 Koppen classification; Af, Am, Aw, Cfa, Csa, Cfb, Cwb (71% of cases), and As, BsH (29% of cases) | Not Specified | 2000 – 2015  5 hottest months each year (city specific) | 90^th^  ≥2 days (1)  ≥3 days (2)  ≥4 days (3)  92.5^th^  ≥2 days (4)  ≥3 days (5)  ≥4 days (6) | Non-heatwave day | Stage 1: quasi-Poisson regression with constrained distributed lag model – 3 degrees of freedom (lag)  Stage 2: city-specific estimates were pooled at the national level using a random-effect meta-analysis with maximum likelihood estimation | Relative humidity  Control for seasonality  Sensitivity analyses - lag from 0–7 days to 0–9 days and df of lag days from three to five. | The 1814 cities were chosen as they had complete hospital data for the study period | 58,400,682  (total) | 0-7 | No increased risk for both 90^th^ and 92.5^th^ percentiles and all heatwave durations |
| IR% = Increased Risk (%) | | | | | | | | | | | |

| **Heat Illness Morbidity - Hospital Admissions** | | | | | | | | | | | |
| --- | --- | --- | --- | --- | --- | --- | --- | --- | --- | --- | --- |
| **Author (year)**  **(reference)** | **Location** | **Population** | **Years of Study** | **Heat Threshold** | **Control Threshold** | **Statistical analysis** | **Confounders Measured** | **Notes** | **Number of Health Events** | **Lags** | **Outcome Measure** |
| Heo  (2019) | South Korea  (7 cities + 9 provinces)  >1 Koppen classification, predominantly Dwa | 50.7 million | 2011 – 2014  Warm season (June - Sep) | 90^th^  2 days  Air Temp (1)  HI (2)  WBGT (3) | Non-heatwave day | Generalized additive model with a link function and a Quasi-Poisson distribution and piecewise regressions and distributed lag nonlinear model – 4df (lags) 2df (time and humidity) | Sensitivity analysis 3df for time | None | Not specified | 0-20 (distributed)  0-1 (moving average) | RR  (1) 1.784 (1.226–2.594)  (2) 2.008 (1.257–3.208)  (3) 1.989 (1.252–3.159)  (1) 2.084 (1.356–3.202)  (2) 2.409 (1.420–4.087)  (3) 2.363 (1.382–4.038) |
| HI = Heat Index; WBGT = Wet-bulb Globe Temperature; RR = Relative Risk (95% CI) | | | | | | | | | | | |

| **Skin Related Morbidity - Hospital Admissions** | | | | | | | | | | | |
| --- | --- | --- | --- | --- | --- | --- | --- | --- | --- | --- | --- |
| **Author (year)**  **(reference)** | **Location** | **Population** | **Years of Study** | **Heat Threshold** | **Control Threshold** | **Statistical analysis** | **Confounders Measured** | **Notes** | **Number of Health Events** | **Lags** | **Outcome Measure** |
| *Zhao  (2019) | Brazil  (1814 cities)  >1 Koppen classification; Af, Am, Aw, Cfa, Csa, Cfb, Cwb (71% of cases), and As, BsH (29% of cases) | Not Specified | 2000 – 2015  5 hottest months each year (city specific) | 90^th^  ≥2 days (1)  ≥3 days (2)  ≥4 days (3)  92.5^th^  ≥2 days (4)  ≥3 days (5)  ≥4 days (6) | Non-heatwave day | Stage 1: quasi-Poisson regression with constrained distributed lag model – 3 degrees of freedom (lag)  Stage 2: city-specific estimates were pooled at the national level using a random-effect meta-analysis with maximum likelihood estimation | Relative humidity  Control for seasonality  Sensitivity analyses - lag from 0–7 days to 0–9 days and df of lag days from three to five. | The 1814 cities were chosen as they had complete hospital data for the study period | 58,400,682  (total) | 0-7 | Small (<5%) significant risk increase across 90^th^ and 92.5^th^ percentiles and heatwave durations |
| IR% = Increased Risk (%) | | | | | | | | | | | |

| **Schizophrenia – Hospital Admissions** | | | | | | | | | | | |
| --- | --- | --- | --- | --- | --- | --- | --- | --- | --- | --- | --- |
| **Author (year)**  **(reference)** | **Location** | **Population** | **Years of Study** | **Heat Threshold** | **Control Threshold** | **Statistical analysis** | **Confounders Measured** | **Notes** | **Number of Health Events** | **Lags** | **Outcome Measure** |
| Yi  (2019) | China  (Hefei)  Koppen classification Cfa | 7.79 million | January 2005 – November 2014 | AT  90^th^  Single Day | 3.3°C (lowest admission rates) | Poisson generalized linear regression model combined with distributed lag non-linear model - natural cubic spline 3df (rainfall, sunshine duration), 7df (time) | Air pollution  (PM_2.5_ and NO_2_)  Rainfall  Sunshine duration  Control for seasonality and day of week  Sensitivity analysis - altering the df for time (6–8 df/year), rainfall (3–5 df) and sunshine duration (3–5 df). | None | 36,607  (total) | All ages  Lags:  0  1  2  0-7  0-14  0-20  Lags:  0  1  2  21-40  Lags:  0  1  2  41-60  Lags:  0  1  2  >60  Lags:  0  1  2 | RR  1.062 (1.019–1.106)  1.056 (1.022–1.090)  1.050 (1.024–1.076)  1.363 (1.170–1.587)  1.179 (0.943–1.474)  1.100 (1.020, 1.230)  1.090 (1.025, 1.180)  1.075 (1.025, 1.040)  1.051 (0.990, 1.120)  1.050 (1.005, 1.095)  1.049 (1.015, 1.080)  1.160 (1.060, 1.275)  1.140 (1.060, 1.220)  1.120 (1.059, 1.080)  1.060 (0.860, 1.300)  1.061 (0.900, 1.250)  1.062 (0.940, 1.200) |
| AT = Apparent Temperature; RR = Relative Risk (95% CI) | | | | | | | | | | | |

| **Alzheimer’s Morbidity - Hospital Admissions and Post-Discharge Deaths** | | | | | | | | | | | |
| --- | --- | --- | --- | --- | --- | --- | --- | --- | --- | --- | --- |
| **Author (year)**  **(reference)** | **Location** | **Population** | **Years of Study** | **Heat Threshold** | **Control Threshold** | **Statistical analysis** | **Confounders Measured** | **Notes** | **Number of Health Events** | **Lags** | **Outcome Measure** |
| Xu  (2019c) | Australia  (Brisbane)  Koppen classification Cfa | Not specified | 2005 – 2013  Post-discharge deaths up to 2 months after discharge | 90^th^  ≥2 days | Non-heatwave day | Case-crossover design with conditional logistic regression. Case-only design used for individual- and community-level characteristics | Air pollution  (PM_10_ and NO_2_)  Relative humidity | None | 907  (total) | 0-7 | Non-signficant OR around 1.25, (all ages) |
| OR = Odds Ratio (95% CI) | | | | | | | | | | | |

| **Injury/Poisoning Morbidity - Hospital Admissions** | | | | | | | | | | | |
| --- | --- | --- | --- | --- | --- | --- | --- | --- | --- | --- | --- |
| **Author (year)**  **(reference)** | **Location** | **Population** | **Years of Study** | **Heat Threshold** | **Control Threshold** | **Statistical analysis** | **Confounders Measured** | **Notes** | **Number of Health Events** | **Lags** | **Outcome Measure** |
| *Zhao  (2019) | Brazil  (1814 cities)  >1 Koppen classification; Af, Am, Aw, Cfa, Csa, Cfb, Cwb (71% of cases), and As, BsH (29% of cases) | Not Specified | 2000 – 2015  5 hottest months each year (city specific) | 90^th^  ≥2 days (1)  ≥3 days (2)  ≥4 days (3)  92.5^th^  ≥2 days (4)  ≥3 days (5)  ≥4 days (6) | Non-heatwave day  See Zhao all-cause morbidity for statistical analysis | Stage 1: quasi-Poisson regression with constrained distributed lag model – 3 degrees of freedom (lag)  Stage 2: city-specific estimates were pooled at the national level using a random-effect meta-analysis with maximum likelihood estimation | Relative humidity  Control for seasonality  Sensitivity analyses - lag from 0–7 days to 0–9 days and df of lag days from three to five. | The 1814 cities were chosen as they had complete hospital data for the study period | 58,400,682  (total) | 0-7 | Small (<5%) significant risk increase across 90^th^ and 92.5^th^ percentiles and heatwave durations |
| IR% = Increased Risk (%) | | | | | | | | | | | |

| **Maternal Morbidity - Hospital Admissions** | | | | | | | | | | | |
| --- | --- | --- | --- | --- | --- | --- | --- | --- | --- | --- | --- |
| **Author (year)**  **(reference)** | **Location** | **Population** | **Years of Study** | **Heat Threshold** | **Control Threshold** | **Statistical analysis** | **Confounders Measured** | **Notes** | **Number of Health Events** | **Lags** | **Outcome Measure** |
| *Zhao  (2019) | Brazil  (1814 cities)  >1 Koppen classification; Af, Am, Aw, Cfa, Csa, Cfb, Cwb (71% of cases), and As, BsH (29% of cases) | Not Specified | 2000 – 2015  5 hottest months each year (city specific) | 90^th^  ≥2 days (1)  ≥3 days (2)  ≥4 days (3)  92.5^th^  ≥2 days (4)  ≥3 days (5)  ≥4 days (6) | Non-heatwave day | Stage 1: quasi-Poisson regression with constrained distributed lag model – 3 degrees of freedom (lag)  Stage 2: city-specific estimates were pooled at the national level using a random-effect meta-analysis with maximum likelihood estimation | Relative humidity  Control for seasonality  Sensitivity analyses - lag from 0–7 days to 0–9 days and df of lag days from three to five. | The 1814 cities were chosen as they had complete hospital data for the study period | 58,400,682  (total) | 0-7 | Small (<2.5%) significant risk increase across 90^th^ and 92.5^th^ percentiles and heatwave durations |
| IR% = Increased Risk (%) | | | | | | | | | | | |

| **Perinatal - Hospital Admissions** | | | | | | | | | | | |
| --- | --- | --- | --- | --- | --- | --- | --- | --- | --- | --- | --- |
| **Author (year)**  **(reference)** | **Location** | **Population** | **Years of Study** | **Heat Threshold** | **Control Threshold** | **Statistical analysis** | **Confounders Measured** | **Notes** | **Number of Health Events** | **Lags** | **Outcome Measure** |
| *Zhao  (2019) | Brazil  (1814 cities)  >1 Koppen classification; Af, Am, Aw, Cfa, Csa, Cfb, Cwb (71% of cases), and As, BsH (29% of cases) | Not Specified | 2000 – 2015  5 hottest months each year (city specific) | 90^th^  ≥2 days (1)  ≥3 days (2)  ≥4 days (3)  92.5^th^  ≥2 days (4)  ≥3 days (5)  ≥4 days (6) | Non-heatwave day | Stage 1: quasi-Poisson regression with constrained distributed lag model – 3 degrees of freedom (lag)  Stage 2: city-specific estimates were pooled at the national level using a random-effect meta-analysis with maximum likelihood estimation | Relative humidity  Control for seasonality  Sensitivity analyses - lag from 0–7 days to 0–9 days and df of lag days from three to five. | The 1814 cities were chosen as they had complete hospital data for the study period | 58,400,682  (total) | 0-7 | Significant risk increase (5 – 10%) across 90^th^ and 92.5^th^ percentiles and heatwave durations increasing with percentile and heatwave duration |
| IR% = Increased Risk (%) | | | | | | | | | | | |

| **Pre-term Birth** | | | | | | | | | | | |
| --- | --- | --- | --- | --- | --- | --- | --- | --- | --- | --- | --- |
| **Author (year)**  **(reference)** | **Location** | **Population** | **Years of Study** | **Heat Threshold** | **Control Threshold** | **Statistical analysis** | **Confounders Measured** | **Notes** | **Number of Health Events** | **Lags** | **Outcome Measure** |
| *Kent  (2014) | United States  (Alabama) | Not specified | 1990 – 2010  Warm season (May – Sep) | 85^th^  1 day (1)  90^th^  1 day (2) | Non-heatwave day | Time stratified case-crossover – natural cubic spline 3df (temperature) | Control for seasonality and day of the week | None | 60,466  (total) | 0 | No significant change in risk 85^th^ percentile. Small (<5%) significant decreased risk 90^th^ percentile |
| Sun  (2019) | United States  (Hot-Humid (1) and Mixed-Humid (2) regions) | Not Specified | 1989 - 2002 | 95^th^ | 50^th^ | Distributed lag non-linear quasi-Poisson time-series model – quadratic b-spline 3df (temperature). Natural cubic b-spline 2df (lags), 3df (day of week, holidays), 8df (time) | Relative humidity  Controlled for day of week and seasonality | The study population was limited to singleton births in the 403 counties with data continuously available throughout the study period | 1,372,985 (total) |  | OR (95% CI)  1) 1.26 (1.26-1.27)  2) 1.06 (1.05, 1.06) |
